# Divergence of gut bacteria through the selection of genomic variants implicated in the metabolism of sugars, amino acids, and purines by small extracellular vesicles in milk

**DOI:** 10.1080/19490976.2025.2449704

**Published:** 2025-01-06

**Authors:** Fang Zhou, Peerzada Tajamul Mumtaz, Haluk Dogan, Roland Madadjim, Juan Cui, Janos Zempleni

**Affiliations:** a Department of Nutrition and Health Sciences, University of Nebraska-Lincoln, Lincoln, NE, USA; b School of Computing, University of Nebraska-Lincoln, Lincoln, NE, USA

**Keywords:** Bacteria, exosomes, extracellular vesicles, genomic variants, milk

## Abstract

Here, we report that small extracellular vesicles (sEVs) in milk mediate the communication between bacteria and animal kingdoms, increase the divergence of bacteria in the intestine, and alter metabolite production by bacteria. We show that bovine milk sEVs select approximately 55,000 genomic variants in 19 species of bacteria from the murine cecum *ex vivo*. The genomic variants are transcribed into mRNA. The selection of genomic variants by milk sEVs alters bacterial metabolism, leading to an up to 12-fold difference in the abundance of more than 1000 metabolites in bacteria cultured in milk sEV-free media compared to sEV-containing media. Evidence is particularly strong that selection of genomic variants by milk sEV changes the metabolism of sugars, amino acids, and purines which might contribute to the development of spatial learning and memory deficiencies and seizure phenotypes reported for milk sEV-depleted infants and mice. Human milk is a rich source of sEVs, whereas formula contains only trace amounts of milk sEVs. This report implicates nutritional sEVs in altered microbial metabolism beyond the mere selection of bacterial communities.

## Introduction

Small extracellular vesicles (sEVs) facilitate cell-to-cell communication and cell-to-environment communication in mammals and gram-negative and -positive bacteria, respectively.^1–3^ In most cases, communication is facilitated by the transfer of regulatory cargo such as small RNAs from sEV donor cells to recipient cells.^4,5^ sEVs and small RNAs do not originate exclusively in endogenous synthesis but are abundant in the diet such as milk.^6,7^ Human milk contains 2.2 *x* 10^11^ sEVs/mL, which harbor more than 200 distinct microRNAs.^7^ An infant consuming 800 mL milk/d^8^ ingests 176 trillion milk sEVs (mEVs) per day. In contrast, infant formulas contain only trace amounts of mEVs and microRNA cargo.^7,9^ Nature has designed milk to be the sole source of nutrition in the early stages of mammalian life. Consistent with an important role of mEVs in the health and development of mammalian offspring, dietary mEV depletion elicits strong phenotypes in infants and neonate mice, including a ninefold decrease in the performance of the Barnes maze test of spatial learning and memory, a fivefold increase in the severity of kainic acid-induced seizures, a 50% decrease in litter size, and a 75% decrease in survival to weaning in mice fed a mEV-depleted diet compared to controls fed a mEV-sufficient diet, as well as changes in the urinary metabolome in formula-fed compared to breastfed infants.^10–12^ Evidence from clinical and epidemiological studies is similar and suggests that dietary depletion of mEVs in formula-fed infants decreases neuronal myelination and brain white matter, as well as general, verbal, and non-verbal cognitive abilities.^13^ mEV supplementation improves intestinal health. For example, supplementation with mEVs decreased the severity of necrotizing enterocolitis by 50% in neonate mice.^14^ These are important considerations in infant nutrition because only 26% of parents in the United States follow the recommendation by the American Academy of Pediatrics to use human milk as the sole source of nutrition in the first 6 months of life.^15,16^ Infants exclusively fed with formula do not realize the benefits of mEVs conferred by breastfeeding.

The gut microbiome may play an important role in transmitting sEV signals in milk to the host. Approximately, 50% of orally administered mEVs escape absorption, survive digestion in the gastrointestinal tract, and reach the large intestine where most of the gut microbiome resides.^9,17,18^ Bacteria internalize mEVs (this study). Consumption of mEV-defined diets is associated with changes in bacterial communities in the murine gut,^19^ and bacterial communities and biosynthetic pathways are different in preterm infants fed mothers’ own milk compared to formula-fed preterm infants.^20^ It is plausible that mEV-dependent changes in bacterial communities in the gut alter both the composition and quantity of metabolites secreted by bacteria and absorbed by the host, a phenomenon referred to as signalome.^21^ Here, we report that the effects of mEVs on the gut microbiome extend beyond the mere selection of bacterial communities, and mEVs select 55,000 genomic variants in 137 genes, including 13 genes in purine metabolism, from 19 bacterial species in cultures of murine cecum extracts compared to reference genomes. The variant genes are transcribed into mRNA, leading to changes in bacterial metabolism. We further report that mEVs elicit an up to 12-fold change in the production of intermediates from purine metabolism, glycolysis and gluconeogenesis, pentose metabolism, and tricarboxylic acid cycle in murine gut bacteria *ex vivo*. We propose that studies of diet-microbiome interactions need to include the selection of genomic variants because the selection of variants drives bacterial divergence and speciation and may affect the health of the host.

Studies were conducted *ex vivo*, as opposed to *in vivo*, based on the following rationale. The data reported here were first generated using samples from mice in a previous study, in which we assessed the effects of mEVs on bacterial communities in the murine gut *in vivo* .^19^ Second, conducting the study *ex vivo* allowed us to tightly control mEV levels in media, which mimicked concentrations in the gut’s aqueous-phase water. Third, we have used monocultures of commensal gut pathogens (*Clostridioides difficile* and *Enterococcus faecalis*) to demonstrate that mEVs select genetic mutations *ex vivo* that make the pathogens less virulent *in vivo*, which speaks to the power of the *ex vivo* approach.^22^

## Results

### Bioinformatics pipelines

We used two complementary bioinformatics pipelines to assess the effects of mEVs on the selection of genomic variants. The MIDAS (Metagenomic Intra-Species Diversity Analysis System) pipeline maps sequencing reads by using 30,000 bacterial reference genomes and reveals the landscape of variants selected by mEVs in species and strains.^23^ The StrainPhlAn pipeline maps sequencing reads by using well-annotated clade-specific marker genes from over ~17,000 bacterial, viral, and eukaryotic reference genomes and allows for a higher level of resolution compared to MIDAS at the strain level.^24^ Analyses were performed by using an average of approximately 130 million raw DNA sequencing reads from each cecum sample cultured in mEV-supplemented (mEVS) and mEV-free (mEVF) media for a total of 400 million reads per treatment group (Supplementary Table S1). After filtering, 367,237,399 and 395,031,435 sequencing reads remained for downstream analysis of mEVS and mEVF cultures, respectively.

### Bovine milk EVs participate in the selection of genomic variants and population structures in mixed cultures of bacteria from the murine cecum

Analysis by MIDAS suggests that bovine milk EVs select genomic variants, including single nucleotide polymorphisms (SNPs), insertions and deletions in anaerobic cultures of cecum content from C57BL/6J mice. Cecum content from the same three mice was aliquoted and added to mEVS or mEVF media to eliminate variations in the input material as confounder. We analyzed DNA-sequencing data by using two levels of stringency, accepting genomic variants that were detected in either all three biological replicates (high stringency) or two replicates (lower stringency).

**Figure 1.**
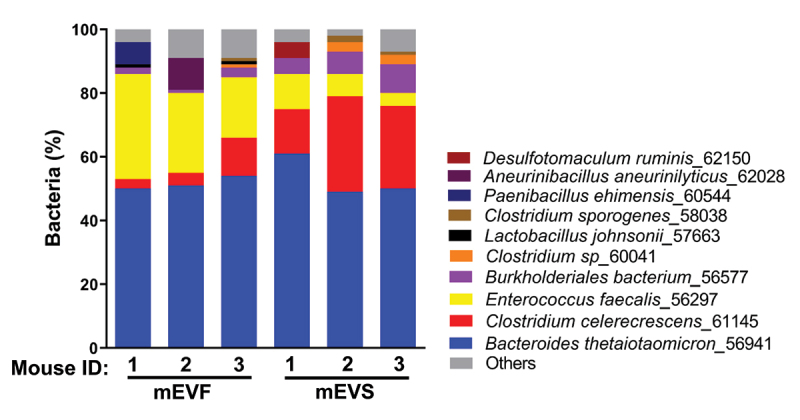
Percent distribution of bacterial species cultured in media defined by the content of milk extracellular vesicles.

More than 200 and 190 million sequencing reads were mapped to 11 and 19 bacterial species in mEVS and mEVF cultures, respectively, by using a threshold of 10× sequencing coverage (Table 1). Seven species were detected in both mEVS and mEVF cultures, four species were unique to mEVS cultures, and 12 species were unique to mEVF cultures. For three of the seven species present in both mEVS and mEVF cultures, their relative abundance differed between cultures: *Clostridium celerecrescens_*61145, *Enterococcus faecalis_*56297, and *Burkholderiales bacterium_*56577 (*p* < 0.05; Figure 1). *C. celerecrescens* was a dominant bacterium because it serves as a reference strain in the MIDAS and StainPhlAn pipelines used in bioinformatics analysis but also represents other Clostridia.

Gut content was collected from ceca of C57BL/6J mice and cultured in sterile M9 minimal salts media supplemented with milk EVs (mEVS) or vehicle (mEVF) under anaerobic conditions for 7 d. Bars represent the percent distribution of bacterial species from three mice per treatment, analyzed by MIDAS. Source data are provided as a Source Data file.

**Table 1.** Bacterial species in mEVS and mEVF cultures.

Variable	mEVS^a^	mEVF
Annotated sequencing reads^b^	207,380,409	191,902,964
Species detected only in mEVS or mEVF^c^	*Anaerotruncus sp_*59696 *Clostridium sp_*57418 *Clostridium drakei_*61273 *Desulfotomaculum ruminis*_62150	*Aneurinibacillus aneurinilyticus*_62028 *Brevundimonas diminuta*_58385 *Clostridium sp_*60561 *Lactobacillus johnsonii*_57663 *Paenibacillus ehimensis*_60544 *Bacillus cereus*_56036 *Lysinibacillus boronitolerans*_54563 *Sporosarcina newyorkensis*_62264 *Clostridium termitidis*_57708 *Lysinibacillus fusiformis*_53142 *Staphylococcus xylosus*_56919 *Paenibacillus barengoltzii*_56244
Species detected in both mEVS and mEVF	*Bacteroides faecis*_58503	
	*Bacteroides thetaiotaomicron*_56941	
	*Burkholderiales bacterium*_56577	
	*Clostridium celerecrescens*_61145	
	*Clostridium sp*_60041	
	*Clostridium sporogenes*_58038	
	*Enterococcus faecalis*_56297	

^a^mEVF, milk extracellular vesicle-free media; mEVS, milk extracellular vesicle-supplemented media.

^b^Values represent the combined total of annotated reads from all three sequencing experiments per treatment. Annotation was achieved by aligning sequencing reads with more than 30,000 bacterial reference genomes in MIDAS.

^c^Species were detected by using MIDAS (*n* = 3per group). Source data are provided as a Source Data file.

**Figure 2.**
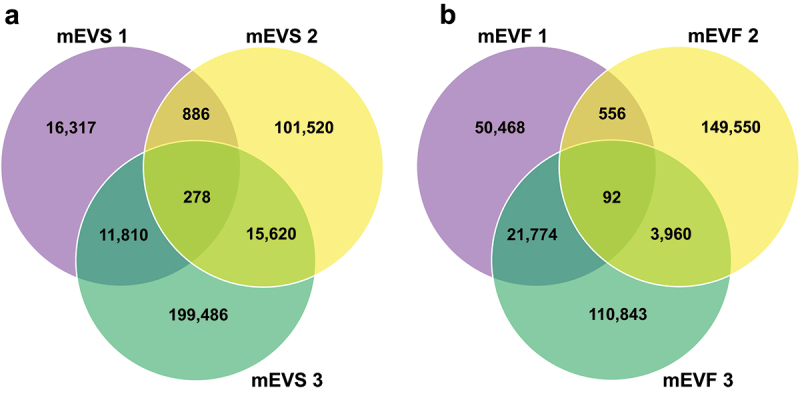
Venn diagrams of genomic variants in bacteria cultured in media defined by the content of milk extracellular vesicles.

In mEVS cultures, 278 and 28,594 strain-level genomic variants were detected in high and low stringency datasets, respectively (Figure 2a). Ninety-five genes carried non-synonymous SNPs in mEVS cultures in the high stringency dataset. In mEVF cultures, 92 and 26,382 strain-level genomic variants were detected in high and low stringency datasets, respectively (Figure 2b). Forty-two genes carried non-synonymous SNPs in mEVF cultures in the high stringency dataset. Genomic variants were slightly underrepresented in coding sequences (CDS) compared to other regions in mEVF cultures of *B. bacterium*_56577 and *E. faecalis*_56297 (Table 2): CDS make up 93% and 90% of the genomes in *B. bacterium*_56577 and *E. faecalis*_56297, respectively, but only 86% and 70% of the genomic variants resided in CDS. In contrast, 87% and 88% of genomic variants resided in CDS in mEVS cultures, and 86% and 70% genomic variants resided in CDS in mEVF cultures of *B. bacterium*_56577 and *E. faecalis*_56297, respectively. In *C. celerecrescens*_61145, 90% and 95% of the genomic variants in mEVF and mEVS cultures, respectively, resided in CDS. Sizes of CDS and whole genomes were accessed through the Integrated Microbial Genomes & Microbiomes system.^25,26^ In some species, the frequency of genomic variants was higher in mEVS cultures compared to mEVF cultures. For example, the number of variants was greater in mEVS cultures of *B. bacterium*_56577 and *E. faecalis*_56297 compared to mEVF cultures (Table 2). The effect of bovine mEVs was particularly pronounced in *E. faecalis*_56297 in which the number of variants was five times greater in mEVS cultures compared to mEVF cultures. In some species, genomic variants clustered in intergenic regions (IGRs). For example, 50% of the genomic variants clustered in IGRs in *Bacteroides thetaiotaomicron*_56941 in both mEVS and mEVF cultures, despite IGRs making up only 2% of the genome in this species (Table 2).

a) Genomic variants in mEVS cultures. b) Genomic variants in mEVF cultures. Gut content was collected from ceca of C57BL/6J mice and cultured in mEVS or mEVF under anaerobic conditions for 7 d. Genomic variants were analyzed by using MIDAS (*n* = 3 mice per group). Aliquots of cecum content from the same mice were used to inoculate mEVS and mEVF cultures, and the numerals after group designations identify the mice. Source data are provided as a Source Data file.

**Table 2.** Genomic variations in CDS or IGR in mEVS or mEVF cultures of murine cecum content.

	mEVS^a^		mEVF	
	CDS	IGR	CDS	IGR
*B. thetaiotaomicron*_56941	2490 ± 239	2475 ± 158	2310 ± 192	2320 ± 88
*B. bacterium*_56577	15,526 ± 2378*	2230 ± 369*	5315 ± 1012	855 ± 144
*C. celerecrescens*_61145	174,986 ± 9828	22,114 ± 740	185,590 ± 17,421	21,559 ± 1213
*E. faecalis_*56297	21,868 ± 1190*	2919 ± 131*	3290 ± 1972	1408 ± 247

^a^CDS, coding sequences; IGR, intergenic regions; mEVF, milk extracellular vesicle-free media; mEVS, milk extracellular vesicle-supplemented media.

*p < 0.05 (mEVS vs mEVF; *n* = 3). Source data are provided as a Source Data file.

Bovine mEVs selected genomic variants in distinct metabolic pathways. Eighteen of the 128 mEV-dependent variants are implicated in the metabolism of sugars, amino acids, and purines in both mEVS and mEVF cultures (Table 3 and Supplementary Table S2).

**Table 3.** Genes with non-synonymous substitutions in *C. celerecrescens*^.a^

	Gene ID^b^	Pathway
mEVS^c^	29354.3.peg.1282	Starch and sucrose metabolism
	29354.3.peg.42; 29354.3.peg.643	Amino sugar and nucleotide sugar metabolism
	29354.3.peg.4350; 29354.3.peg.858	Glycine, serine and threonine metabolism
	29354.3.peg.64	Arginine and proline metabolism
	29354.3.peg.2509	D-Glutamine and D-glutamate metabolism
	29354.3.peg.2075; 29354.3.peg.2043; 29354.3.peg.3018	Porphyrin and chlorophyll metabolism
	29354.3.peg.292	Pantothenate and CoA biosynthesis
	29354.3.peg.2621	Oxidative phosphorylation
	29354.3.peg.1970	Peptidoglycan biosynthesis
mEVF	29354.3.peg.20	Purine metabolism
	29354.3.peg.1435	Tryptophan metabolism
	29354.3.peg.59	Arginine and proline metabolism
	29354.3.peg.1765	Glycerophospholipid metabolism
	29354.3.peg.1203	Glycolysis and gluconeogenesis

^a^Non-synonymous substitutions were detected in all three independent cultures.

^b^IDs as per the Pathosystems Resource Integration Center 3.6.8 (https://www.patricbrc.org/).

^c^mEVF, milk extracellular vesicle-free media; mEVS, milk extracellular vesicle-supplemented media.

Source data are provided as a Source Data file.

Bovine mEVs altered the relative copy number (RCN) of genes. At the level of high stringency, RCNs of four and six genes were higher in *B. thetaiotaomicron*_56941 and *B. bacterium*_56577, respectively, cultured in mEVF compared to mEVS media (Supplementary Figure. S1a). In contrast, RCNs of three genes were lower in *E. faecalis*_56297 cultured in mEVF compared to mEVS. At the level of low stringency, additional RCNs were detected in two biological replicates. RCNs of 17 genes were higher in *Clostridium sp*_60041 cultured in mEVF compared to mEVS when using a twofold change as cutoff (Supplementary Figure. S1b). In contrast, the RCNs of 228 genes were higher in *Clostridium sp*_60041 cultured in mEVS compared to mEVF (see Supplementary Figure. S1 for representative examples). Collectively, bovine mEVs selected both bacterial communities and genetic variants in the murine gut microbiome.

### Bovine mEVs select genomic variants in marker genes in bacterial strains

**Figure 3.**
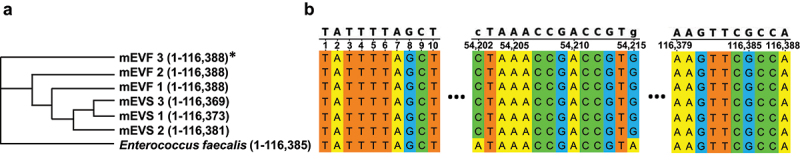
Genomic variants in *E. faecalis* cultured in media defined by the content of milk extracellular vesicles.

Variants were also identified when focusing on marker genes using the StrainPhlAn pipeline.^24^ Eight bacterial strains were detected by using the StrainPhlAn pipeline, and seven of the strains matched those identified by using MIDAS (Supplementary Table S3). The only exception is *Oscillibacter sp*. 1–3, which was unique to the StrainPhlAn pipeline. Three of the eight species satisfied the recommended minimum of detecting 50% of the marker genes in their clades in StrainPhlAn analysis. We detected a total of 6,715 genomic variants across all loci in the three species for both mEVS and mEVF cultures combined, including 6,694 variants in protein coding regions [5,182 non-synonymous (77%) and 1,512 synonymous (23%) variants]. The non-synonymous variants included 62 insertions and 75 deletions. Non-synonymous variants occurred more frequently in *Clostridium sporogenes* than in other species (Supplementary Table S4). Divergence of species was observed in phylogenomic trees in both mEVS and mEVF cultures albeit with a species-specific bias toward one of the culture conditions. For example, genomic divergence was greater in mEVF compared to mEVS cultures in *E. faecalis* and *C. sporogenes* (Figure 3; Supplementary Figure. S2a, b), whereas divergence of *Lactobacillus johnsonii* was observed only in mEVF cultures (Supplementary Figure. S2c, d). The number of genomic variants was sufficiently high to allow for a phylogenomic analysis in only four out of six samples each for *C. sporogenes* and *L. johnsonii*.

a) Phylogenomic tree of marker genes in *E. faecalis*. b) Multiple sequence alignment of concatenated *E. faecalis* markers from mEVS and mEVF cultures with the *E. faecalis* reference genome (RefSeq: GCF_902161805.1). Gut content was collected from ceca of C57BL/6J mice and cultured in mEVS or mEVF under anaerobic conditions for 7 d (*n* = 3 mice per group). The phylogenomic tree was built using the RAxML algorithm. *Numbers in parentheses identify the length of the concatenated markers. Sequencing reads of marker genes were aligned with the reference sequence in the MetaPhlAn2 database. Source data are provided as a Source Data file.

**Figure 4.**
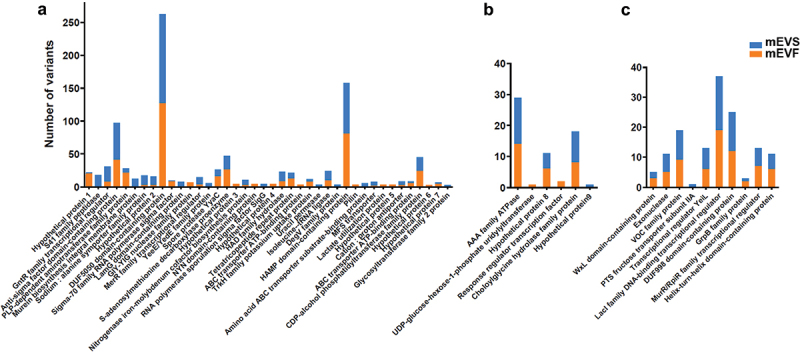
Number of genomic variants in strains from three bacterial species strains cultured in media defined by the content of milk extracellular vesicles.

Some strains and genomic loci were more susceptible than others to accumulating genomic variants in protein-coding genes. Genomic variants were identified in 56 protein-coding genes in *C. sporogenes* (40 unique genes), *L. johnsonii* (6) and *E. faecalis* (10) (Supplementary Table S5). Five hundred-eighteen out of 1007 variants in *C. sporogenes* resided in only three genes: a gene coding for a protein of unknown function containing a DUF5050 domain (DUF5050 domain-containing protein), a gene coding for a protein containing a HAMP domain (HAMP domain-containing protein), and a gene coding for a protein containing an anti-sigma factor domain (anti-sigma factor domain-containing protein) (Figure 4a). In *L. johnsonii*, 58 genomic variants (94% of total) were identified in genes coding for a member of the superfamily of ATPases associated with diverse cellular functions (AAA family ATPase), a protein from choloylglycine hydrolase (choloylglycine hydrolase family protein) and a hypothetical protein (Figure 4b). In *E. faecalis*, genomic variants were detected in 10 protein-coding genes. Fifty-nine percent of the genomic variants in *E. faecalis* were identified in three genes (Figure 4c): a gene coding for a protein of the lactose operon repressor (LacI) family of transcriptional regulators (LacI family DNA-binding transcriptional regulator), a gene coding for a protein of unknown function from a family of conserved archaeal proteins containing a DUF998 domain (DUF998 domain-containing protein), and a gene coding for a protein from the superfamily of vicinal oxygen chelators (VOC family protein). Genomic variants in these genes were detected in both mEVS and mEVF cultures. Collectively, we observed that some bacterial strains were more resistant to the selection of genetic variants than others, and insertions and deletions were rare events.

a) *Clostridium sporogenes*. b) *Lactobacillus johnsonii*. c) *Enterococcus faecalis*. Gut content was collected from ceca of C57BL/6J mice and cultured in mEVS or mEVF media under anaerobic conditions for 7 d (*n* = 3 mice per group). Blue bars represent the number of variants in mEVS; orange bars represent the number of variants in mEVF. Source data are provided as a Source Data file.

In some genes, variants were detected in only one of the two culture conditions. For example, seven and nine genes genomic variants were detected only in mEVS and mEVF cultures, respectively, in *C. sporogenes* (Supplementary Table S5). All 13 variants in the gene coding for murJ (murein biosynthesis integral membrane protein) were identified in mEVS cultures in *C. sporogenes*. Variants in genes coding for a type II secretion system protein (pilin) and a protein related to the utilization of lactate (lactate utilization protein) only were detected in mEVF cultures in *C. sporogenes* (Figure 4a). KEGG pathway analysis implicated synonymous and non-synonymous genomic variants in *C. sporogenes* in regions coding for enzymes implicated in purine metabolism and biosynthesis of cofactors, such as nicotinamide adenine dinucleotide (NAD) and pantothenate biosynthesis in mEVS cultures and aminoacyl-tRNA biosynthesis in mEVF cultures (Supplementary Table S6).

### Transcription of genomic variants

Forty-eight to 70 million raw reads were obtained for each of 12 samples (two cultures × two sexes × 3 replicates) by RNA sequencing analysis using a 100-bp paired-end protocol. For mEVS and mEVF cultures 55,894,867 ± 6,335,845 and 61,127,337 ± 4,910,738 reads, respectively, passed quality checks (Q score > 10 and length ≥ 15bp) and were included in subsequent analyses (Supplementary Table S7, Table 1). When the reads were mapped using the MIDAS pipeline, we detected 63,613 and 56,385 RNA-level variants in 9,165 and 6,914 unique transcripts in 12 and 9 bacterial species mEVS and mEVF cultures, respectively. Transcripts from bacteria in female mice harbored significantly less variants in mEVF and mEVS cultures, respectively, than male mice. Three thousand six hundred and thirty-seven and 3,683 transcripts matched synonymous and non-synonymous genomic variants in mEVF and mEVS cultures, respectively, in the low stringency dataset (Supplementary Table S7, Tables 2 and 3). Six hundred-eight and 1,494 transcripts matched synonymous and non-synonymous genomic variants in mEVF and mEVS cultures, respectively, in the high stringency dataset. When only non-synonymous variants were considered, 39 and 215 variant transcripts matched genomic variants in mEVF and mEVS, respectively, in the high stringency dataset. In both mEVS and mEVF cultures, variants clustered in pathways of glucose metabolism, energy homeostasis and metabolism of cofactors (purines) in glucose and energy homeostasis, which accounted for 52% and 63% of all variants detected in mEVS and mEVF cultures, respectively (*p* < 0.05; Supplementary Figure. S3, Supplementary Table S7, Tables S4 and S5). Collectively, genetic variants are transcribed and cluster in pathways implicated in major metabolic pathways.

### Bacterial metabolome

**Figure 5.**
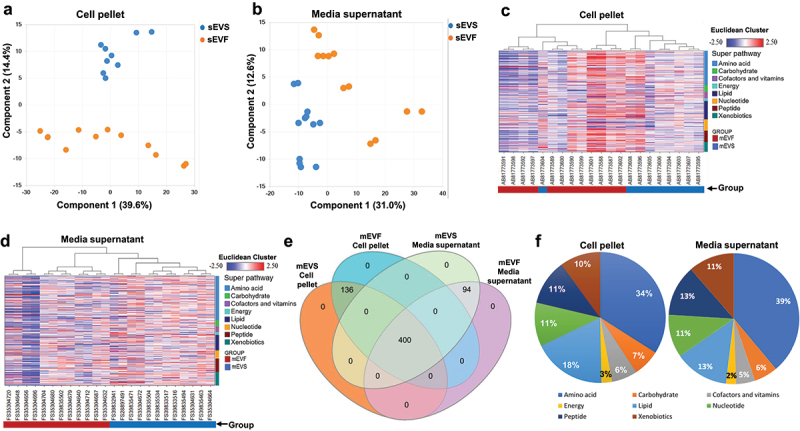
Bacterial metabolites in cultures defined by the content of milk extracellular vesicles. Gut content was collected from ceca of C57BL/6J mice and cultured in mEVS or mEVF media under anaerobic conditions for 7 d (*n* = 3 mice per sex and group).

The selection of genomic variants was associated with changes in the bacterial metabolome. We assessed the bacterial metabolome in both bacterial pellets and cell-free media supernatant by using non-targeted metabolomics analysis. Bacterial cultures from the ceca of both male and female mice were analyzed. We identified a total of 536 and 494 mEV-dependent metabolites in cell pellets and media supernatant, respectively, for both sexes and media combined. Ninety and 147 metabolites were significantly different in cell pellets and media supernatants, respectively, from mEVS and mEVF cultures. The abundance of metabolites was distinct for the two media, as per principal component analysis (PCA) and hierarchical cluster analysis (HCA; Figure 5a-d). Most metabolites were shared across treatment groups and input material (Figure 5e). Exceptions included 136 metabolites that were detected only in cell pellets from mEVS cultures and 94 metabolites that were detected only in media from mEVF cultures. mEV-dependent metabolites were overrepresented in distinct super pathways, including the metabolism of amino acids, lipids, carbohydrates, and nucleotides (Figure 5f, Supplementary Tables S8 and S9).

a) Principal component analysis of metabolites in cell pellets. Plots represent the combined total of all metabolites in both male and female mice cultured in mEVS and mEVF media (*n* = 12). b) Principal component analysis of metabolites in cell-free media supernatants. Plots represent the combined total of all metabolites in both male and female mice cultured in mEVS and mEVF media (*n* = 12). c) Hierarchical clustering analysis of metabolites in cell pellets significantly altered by the content of mEVs in bacterial cultures. d) Hierarchical clustering analysis of metabolites in cell-free media supernatant significantly altered by the content of mEVs in bacterial cultures. e) Venn diagram of metabolites significantly altered in bacterial pellets and cell-free media supernatant from mEV-defined bacterial cultures. f) Percent distribution of metabolites significantly altered by mEV content among super pathways in cell pellets and cell-free supernatant.

### Genomic variants in the metabolism of purines and sugars

For an in-depth analysis of the association between genomic variants and changes in bacterial metabolism, we focused on pathways implicated in the metabolism of purines and sugars based on the following rationale. First, the dietary intake of milk sEVs altered the levels of purine metabolites in urine and tissues in infants, adults, and mice.^27^ Second, purine compounds such as ATP, cAMP, GTP, NAD and NADP play crucial roles in energy metabolism and purinergic receptor signaling in the brain.^28–32^ Third, alterations in the metabolism of purines and sugars by gut bacteria under mEV-defined conditions are consistent with a report that spatial learning and memory were impaired and the severity of kainic acid-induced seizures increased in mice fed an mEV-depleted diet compared to controls fed an mEV-sufficient diet.^10^ Fourth, the metabolism of sugars and purines ranked among the top five mEV-dependent super pathways.

mEV-dependent genomic variants were overrepresented in pathways of purine metabolism. Thirty-nine of the 338 genes harboring 325 genomic variants identified by StrainPhlAn were implicated in purine and nucleic acid metabolism (Supplementary Table S7, Table S5). Genomic variants were more frequent in the following five genes than in any other gene that harbored polymorphisms: DNA polymerase I (EC 2.7.7.7), phosphoribosylformylglycinamidine synthase (EC 6.3.5.3), ribonucleoside-triphosphate reductase (EC 1.17.4.2), DNA polymerase III (EC 2.7.7.7), DNA polymerase III polC-type (EC 2.7.7.7) (Supplementary Table S7, Table S5). Six variants of genes in purine metabolism were detected at the genome level, and four variants were detected at the transcript level in *Clostridium celerecrescens* 152B and *C. sporogenes*. Three variants of genes in purine metabolism [adenosine deaminase (*add*), adenine deaminase (*ade*), and N5-carboxyaminoimidazole ribonucleotide mutase (*purE*)] were detected at both the genome and transcript level and represent a particularly high level of confidence.

**Figure 6.**
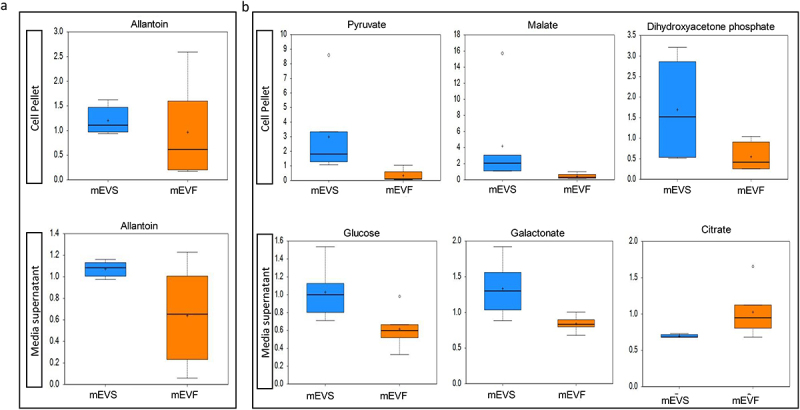
Levels of purine, energy and sugar metabolites in cell pellets and media supernatants from bacterial cultures in media defined by the content of milk extracellular vesicles.

mEV-dependent changes in genomes and transcriptomes were associated with changes in the metabolome. We identified 26 purine metabolites in bacterial pellets and 21 in media supernatants. However, the effect of mEVs was statistically significant only for allantoin which was 2.2-fold and 2.0 higher in media and cell pellets, respectively, from mEVS cultures compared to mEVF cultures (*p* < 0.05; Figure 6a). Allantoin is an end product in bacterial purine catabolism (Supplementary Figure. S4; Supplementary Table S9). Changes in purine metabolites were associated with changes in metabolites from major pathways of sugar and energy metabolism. We identified 36 and 29 sugar metabolites and intermediates from energy metabolism in bacterial pellets and media supernatants. The levels of 24 metabolites were up to 3.7-fold different in pellets and media from mEV-defined cultures (*p* < 0.05; Figure 6b; Supplementary Table S9). The compounds are intermediates in glycolysis, gluconeogenesis, Krebs cycle, and pentose phosphate pathway (Supplementary Figure S5).

a) Levels of significantly altered purine metabolite in cell pellets and media supernatants (*p* < 0.05). b) Levels of significantly altered energy and sugar metabolites in cell pellets and media supernatants (*p* < 0.05). The y-axes are the relative metabolite concentrations. The central horizontal lines in boxes represent median values, and + denotes mean values; the top and bottom of boxes represent upper and lower quartiles. Error bars depict the minimum and maximum distributions, whereas ○ identifies extreme data points.

### Bacteria internalize mEVs

**Figure 7.**
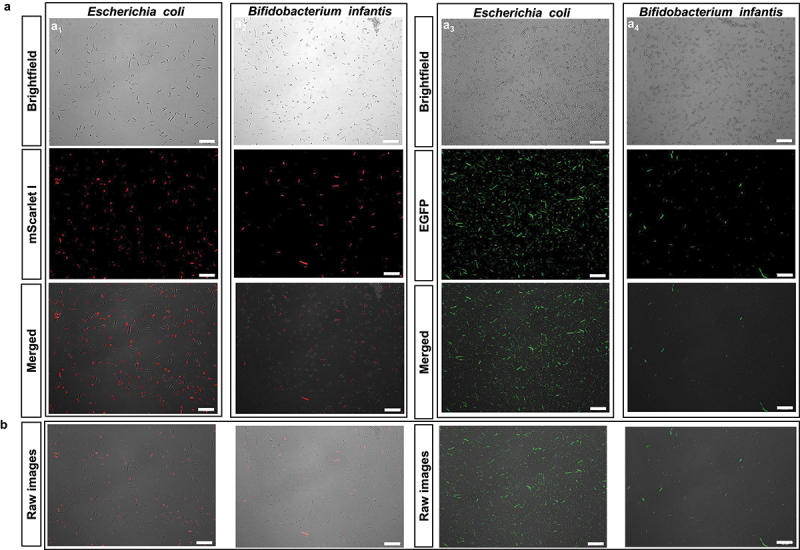
a) Expression of plasmids encoding EGFP and mScarlet-i, loaded into and delivered by mEvs, in B. infantis and E. coli.

Bacteria internalized mEVs, which is consistent with the selection of genomic variants by mEVs. In a first round of experimentation, we added HiLyte 750-labeled mEVs at nutritionally relevant concentrations to cultures of Gram-positive *B. subtillis* and Gram-negative *E. coli*. The internalization of mEVs by bacteria reached a plateau within less than 10 min (Supplementary Figure S6a). However, the data do not allow to distinguish between adsorption of mEVs to the bacterial surface and internalization of mEVs by bacteria. This limitation was addressed in a second round of experimentation, in which we used the expression of reporter plasmids as readout. mEVs were loaded with plasmids pMRE-Tn7–135 (encoding mScarlet I) or pBAV1K-T5-gfp (encoding EGFP) and added to cultures of *E. coli* and Gram-positive *Bifidobacterium infantis*. The expression of fluorescent proteins was assessed using confocal microscopy and fluorescence activated cell sorting (FACS). We detected mScarlet I and EGFP in both bacterial species (Figure 7; columns a_1_ and a_2_ denote mScarlet I, and columns a_3_ and a_4_ denote EGFP). The images depict protein expression with (Figure 7a) and without (Figure 7b) electronically enhanced brightness and contrast. Data from confocal microscopy were independently confirmed using FACS. In FACS analysis, control cells from both bacterial strains formed distinct populations, with forward scatter (FSC) as the discriminant to exclude background noise (Supplementary Figure S6b_1_-b_2_). Bacteria treated with pBAV1K-T5-gfp – loaded mEVs expressed EGFP, while mScarlet I fluorescence was not detectable (Supplementary Figure S5b_3_-b_4_). Bacteria treated with pMRE-Tn7–135–loaded mEVs expressed mScarlet I, while EGFP fluorescence was not detectable (Supplementary Figure S6b_5_-b_6_).

The images were captured using laser scanning confocal microscopy 6 h after induction by IPTG and L-arabinose. Each image was captured through a 60× magnification lens. Scale bar: 20 µm. b) Same as panel a, but without enhancing brightness and contrast. It serves as a reference point, allowing for a direct comparison with the enhanced images in panel (a1-4), which underwent specific brightness and contrast enhancements.

## Discussion

This report links dietary EVs with the selection of genomic variants in gut bacteria. Previous studies, including ours, were limited in scope by focusing on EV-dependent changes in bacterial communities in the gut.^19,33^ This report is important because it implicates mEVs in the divergence of gut bacteria as a first step in speciation and altered bacterial signalome in the host.^21,34^ There is precedent for diet-driven selection of genomic variants and mutations. For example, the −13910*T mutation in the *lactase* gene leads to persistent expression of lactose in adults and conferred a growth advantage to European populations practicing dairy farming.^35^ The selection of a mutation in the *lactase* gene by dietary lactose does not distract from this report’s novelty because here we show for the first time that a *bona fide* signal transmitter from the animal kingdom, mEVs, communicates with the bacteria kingdom leading to genomic divergence in bacteria. Both Gram-positive and Gram-negative bacteria communicate with the environment through bacterial EVs.^36,37^ Our observation that bacteria internalize bovine mEVs is consistent with EV-dependent communication in bacteria, but that does not exclude the possibility that some bacteria degrade mEVs and are auxotrophic for the degradation products. The roles of mEVs in bacterial signaling and nutrition are not mutually exclusive.

The selection of genomic variants by mEVs was achieved using a nutritionally relevant concentration of mEVs in culture media within only 7 d of culturing, *i.e.*, this report has implications for human nutrition and health. We previously reported that dietary depletion of mEVs elicits an increase in the concentration of purine metabolites in body fluids and tissues from adults, infants and mice.^27^ In the light of the new discoveries reported here, we speculate that altered bacterial metabolism of purines in the gut might have contributed to the increase in purine metabolites observed in humans and mice on mEV-depleted diets. For example, the c.562C>A variation in the *ade* gene (adenine deaminase, EC 3.5.4.2) in *C. celerecrescens* changes an amino acid change in the substrate binding site of the enzyme.^38^ However, this is an untested hypothesis and requires experimental confirmation, especially when considering that the amino acid substitution constitutes a conservative change (*p.Leu188Ile*). We further speculate that bacterial metabolites such as short chain fatty acids, tryptophan, and bile acid derivatives might play a role in decreasing the severity of necrotizing enterocolitis in mEV-supplemented mice.^14,39^ Potentially rewarding lines of future investigation include changes in antibiotics resistance and virulence in pathogenic bacteria. For example, single point mutation in *Clostridium difficile* ribotype RT027, selected by trehalose, increased the virulence of this commensal gut pathogen.^40^

This study was conducted using mature cow’s milk from a local grocery store for preparing mEVs. Using milk from a grocery store has the advantage that milk from many cows is pooled at the producer’s end, thereby eliminating intra-individual variation as a confounder. The trade-off is that one does not know breed, parturition, and age of the cow. mEVs from different breeds and lactational stages might have unique effects on the gut microbiome and host. For example, the mEV cargo differed between Holstein and Normande breeds, and colostrum had a strong protective effect against necrotizing enterocolitis than mature milk in intestinal organoids.^41,42^ We propose that mEVs from distinct mammalian species might also have unique effects on the gut microbiome, but this is an untested hypothesis. Studies are ongoing in our laboratory to assess the effects of human mEVs on the communities, genetic variants, and metabolism in bacteria from infant feces. The ongoing studies will enable to assess the relevance of the selection of genomic variants in a population at risk for mEV depletion.

Among human cohorts, this report is relevant in infant nutrition because most infants born in the United States are partially or exclusively formula-fed, and the formula contains only trace amounts of mEVs.^7,15,16^ This is a concern because consumption of an mEV-depleted diet impaired brain health and development in neonate mice.^10^ The significance of this report may extend beyond mEVs and includes the selection of genomic variants by plant foods, which also contain EVs.^33,43^

We acknowledge the following limitations and uncertainties of this report. While there is good consensus regarding the bacterial species detected by MIDAS and StrainPhlAn, the genomic variants revealed different mEV-dependent pathways when analyzed by the two pipelines. This can be attributed to differences in gene coverage in MIDAS compared to StrainPhlAn.^23,24^ We expect that additional variants will emerge as pipelines for mapping the variants improve. We did not formally assess the mechanisms leading to the divergence in evolving bacterial populations, as well as bacterial communities in distinct regions of the gut. A previous report suggests that multiple mechanisms contribute to diversity in glucose-limited cultures and the capacity of a population to diversify is a function of growth rate.^44^ Ongoing studies focus on mEV-dependent checkpoints of bacterial divergence, associations of genomic variants with changes in transcriptome and metabolome, and phenotypes of bacterial divergence in the host.

## Methods

### Ethical statement

All animal care procedures and experimental procedures were conducted following the protocols approved (protocol 1229) by the Institutional Animal Care Program at the University of Nebraska-Lincoln.

### Mice

C57BL/6J mice aged 7 weeks were purchased from the Jackson Laboratory (stock no. 000664) and acclimated for 7 d (22°C, 12-h light/dark cycle) with free access to food (2016 Teklad global 16% protein rodent diet; Teklad 2916, Envigo, Inc., Madison, WI, USA), and water prior to collection of cecal content.

### Collection and culture of cecal content

Mice were euthanized by using carbon dioxide followed by cervical dislocation, and cecal content was immediately suspended in 10 mL of anaerobic sterile M9 minimal salts media (MM; Gibco Life Technologies, Carlsbad, CA, USA) supplemented with 2 mm MgSO_4_, 0.1 mm CaCl_2_ and 5 mL of sterile-filtered gut content in MM media using a BD GasPak^TM^ EZ anaerobe container system (Becton – Dickinson, Sparks, MD, USA) at room temperature. Aliquots of ceca content were cultured in mEVS and mEVF media. For preparation of mEVS media, sEVs were isolated from store-bought skim bovine milk using sequential ultracentrifugation, sterile-filtered using a 0.22-µm filter, and suspended in phosphate-buffered saline (PBS).^45^ mEVs were authenticated using Nanosight NS300 nanoparticle size analysis, immunoblot analysis, and scanning and transmission electron microscopy as previously described.^19^ Protocols have been deposited in the open-access EV-Track database (ID EV210338). mEVS and mEVF cultures were prepared by adding mEV suspension and PBS, respectively, to media. The concentration of mEVs in mEVS cultures was the equivalent of mEVs from 0.5 L bovine milk distributed in the intestinal water space in a human adult, normalized by body weight for mice.^46^

Samples intended for use in metagenomics analysis were cultured in an anaerobic chamber (Coy Laboratory Products Inc., Grass Lake, MI, USA) at 37°C for 7 d and bacteria were pelleted by centrifugation for isolation of DNA (5,000 × g, 10 min, room temperature). Samples intended for use in metabolomics and transcriptomics analysis were cultured in *Gut microbiota medium* (GMM) (Supplementary Table 10).^47,48^ Three mice were used for each treatment, and media inoculated with PBS was used as control. Cultures were collected after 7 d, pelleted by centrifugation, and the media supernatant was filter-sterilized using 0.2 µm filter. Both the media supernatants and bacterial cell pellets were flash frozen in liquid nitrogen and stored at −80°C until analysis.

### Metagenomics and mRNA transcriptomics analyses

DNA was extracted using the PowerSoil DNA Isolation Kit following the manufacturer’s instructions (Mo Bio Laboratories Inc., Carlsbad, CA, USA). The purity and integrity of DNA were assessed by using the 260-to-280 nm ratio (Nanodrop ND-1000; Nanodrop Technologies, Wilmington, DE, USA) and agarose gel electrophoresis (0.8%). Libraries were prepared by using the Nextera XT kit (Illumina, San Diego, CA, USA) and their quality was assessed using an Agilent Bioanalyzer 2100 (Agilent Technologies, Santa Clara, CA, USA) in the Next Generation Sequencing Core Facility at the University of Nebraska Medical Center. Samples were sequenced using the Illumina NextSeq 500 platform (Illumina, San Diego, CA, USA) using a 75-basepair (bp) single end protocol with an estimated coverage of 150×. We used Trimmomatic to remove reads with a Phred quality score of less than Q30, suggesting that less than 1 in 1000 base calls were incorrect in the remaining reads.^49^

Two bioinformatics pipelines were used to identify genomic variants selected by mEVs. We used the MIDAS pipeline to reveal the landscape of species abundance and strain-level genomic variants by alignment against more than 30,000 bacterial reference pangenomes. In addition, MIDAS was used to assess species-level extent of population structure among the metagenomic data and quality pan-genome gene content for prevalent bacterial species.^23^ We used the StrainPhlAn pipeline to identify dominant sequence variants by concatenated alignment of species-specific marker genes at the strain level, thereby leading to a higher level of resolution compared to MIDAS.^24^ StrainPhlAn uses the same reference library as MetaPhlAn2, which includes clade-specific marker genes from ~13,500 bacterial and archaeal, ~3,500 viral and ~110 eukaryotic reference genomes.^50^ Muscle was used to call variants in MetaPhlAn2 marker genes through StrainPhlAn.^51,52^ We required a minimum of 50% of markers to be present in clades for each sample. Phylogenomic trees of strains were built based on the reconstructed marker genes by using the Randomized Axelerated Maximum Likelihood (RAxML) algorithm.^34^ KEGG pathway analysis was performed by using a hypergeometric test.^53^ The Integrated Microbial Genomes & Microbiomes system was used to assess the sizes of CDS and whole genomes in *B. bacterium*, *C. celerecrescens* and *E. faecalis*.^25,26^

RNA was extracted from cell pellets of three independent biological replicates for each sex and media using RNeasy Mini Kit (Qiagen), following the manufacturer’s recommendation. The RNA concentration and quality were determined using Qubit analyzer, and the Agilent RNA ScreenTape assay in combination with the TapeStation system. Analysis of data generated was performed using the Agilent TapeStation software 4.1. Total RNA yield from the samples ranged from 44 to 200 ng/ul. Subsequently, the ribosomal RNA was removed using the QIAseq FastSelect −5S/16S/23S rRNA removal kit (Qiagen), designed to selectively remove 5S, 16S, and 23S rRNA from complex bacterial community samples. The rRNA-free samples (100 ng) were used for cDNA library preparation and purification using KAPA RNA Hyperprep Kit (Roche). Each resulting uniquely dual-indexed library was quantified and quality accessed by Qubit and Agilent TapeStation, and multiple libraries were pooled in equal molarity. The pooled libraries were sequenced with 100-bp paired-end configuration on an Illumina NovaSeq 6000 sequencing platform. Transcript variants were linked with metabolic pathways using the Bacterial and Viral Bioinformatics Resource Center database.^54^

### Metabolomics analysis

Global metabolomics profiling was conducted using ultra-high-performance liquid chromatography-tandem mass-spectrometry by Metabolon Inc. (Morrisville, NC) as previously described.^55,56^ Briefly, samples were prepared using MicroLab STAR (Hamilton; Reno, NV). Proteins were precipitated with methanol under vigorous shaking for 2 min and removed by centrifugation using GenoGrinder 2000 (Glen Mills; Clifton, NJ). Supernatants were analyzed by reverse phase ultra-high-performance liquid chromatography – tandem mass spectroscopy analysis (RP)/UPLC-MS/MS methods with positive ion mode electrospray ionization (ESI), RP/UPLC-MS/MS with negative ion mode ESI, and hydrophilic interaction liquid chromatography/ultra-high-performance liquid-chromatography-tandem mass spectroscopy (HILIC/UPLC-MS/MS) with negative ion mode ESI. Samples were analyzed using a ACQUITY UPLC (Waters Corporation; Milford, MA) and Q-Exactive high-resolution/accurate mass spectrometer interfaced with a heated ESI source and Orbitrap mass analyzer operated at 35,000 mass resolution (Thermo Fisher Scientific; Waltham, MA). The raw data was processed using Metabolon’s hardware and software as previously described.^56,57^ Metabolites were identified by comparison with libraries of authenticated standards with known retention time/indices, mass to charge ratios, and chromatographic and MS/MS spectral data. Biochemical identification was based on mass match (±10 ppm), retention index, and forward- or reverse-search matching between the experimental data and library standards. The consistency of peak identification and library matches for each compound were checked for each sample and corrected if necessary. Peaks were quantified using area-under-the-curve. Peak area values allowed the determination of relative quantification of metabolites among samples.^58^

### Internalization of bovine mEVs by bacteria

#### Bacterial strains and media

*Bacillus subtilis* 168 (ATCC 23,857) and *Bifidobacterium longum* subsp. *infantis* (ATCC 15,697) were obtained from the American Type Culture Collection (Manassas, VA). *Escherichia coli* DH5α was purchased from Thermo Fisher Scientific (Waltham, MA). *Bacillus subtilis* and *E. coli* were cultivated in LB (10 g/L tryptone, 5 g/L yeast extract, 10 g/L NaCl) at 37°C under aerobic conditions in flasks with shaking at 200 rpm. *Bifidobacterium infantis* was grown in de Man Rogosa Sharpe broth [MRS broth (MilliporeSigma; Burlington, MA)] supplemented with 0.05% (w/v) cysteine (Sigma-Aldrich, St. Louis, MO and 2% (w/v). In reporter plasmid studies, media were supplemented with arabinose (0.1%) and isopropyl ß-D-1-thiogalactopyranoside [IPTG, (0.5 mm)]. *B. infantis* was anaerobically grown in a vinyl chamber (Coy Laboratory Products, Grass Lake, MI) at 37°C for 24 h, in an atmosphere consisting of 5% carbon dioxide, 5% hydrogen, and 90% nitrogen. Optical density was assayed using a using Biotek Synergy H1m microplate reader (BioTek Instruments, Inc.; Winoosky, VT).*Uptake of fluorophore-labeled mEVs*

Milk EVs were isolated from skim bovine milk by differential ultracentrifugation and labeled with carbonyl-reactive HiLyte^TM^ Fluor 750 hydrazide (AnaSpec, Inc, AS-81268) as previously described.^59^ We incubated *Bacillus subtilis* 168 (ATCC 23,857) and *Escherichia coli* with HiLyte 750-labeled mEVs and assessed their uptake using an Odyssey Imaging System (LI-COR Bioscience; Lincoln, NE). Bacteria were incubated using an mEV concentration equivalent to mEVs from 0.5 L bovine milk suspended in the total gastrointestinal water in a human adult (1.7 × 10^10^/mL).

#### Uptake of plasmid-loaded mEVs

Five micrograms of plasmid [addgene #26702 (EGFP) or addgene #118553 (mScarlet-I)] were added to 50 μL mEV suspension, corresponding to ∼3 × 10^8^ mEVs, in a final volume of 50 μL electroporation buffer (Bio-Rad #1652677). The suspensions were mixed and incubated for 5 min at room temperature. Electroporation was carried out using 0.1-cm electroporation cuvettes (Bio-Rad 1,652,083) in a GenePulser Xcell electroporator (Bio-Rad; Hercules, CA). Electroporation was carried out at 1000 V and 125 μF with 5 pulses and 10-s intervals between pulses. Free plasmids were removed by treating samples with DNase I (1.5 Kunitz units/μL, Qiagen #79254), followed by washing with PBS, centrifugation at 130,000 *g* at 4°C, and resuspending the mEVs in 50 μL PBS.

Plasmid-loaded mEVs were added to bacterial cultures in 1–2 mL of antibiotics-free media and incubated at 37°C for 2 h in a shaking incubator under aerobic or anaerobic conditions. Clones were selected on LBS or MRS agar plates with antibiotics [ampicillin (100 μg/mL) or kanamycin (50 μg/mL)]. Clones were grown in liquid media until the absorbance at 600 nm reached 0.5–0.6 units. Expression of EGFP and mScarlet-I was induced with 0.5 mm IPTG and 0.1% L-arabinose at 37°C for 6 h. For analysis by confocal laser scanning microscopy, bacterial cultures were centrifuged at 2,500 × *g* for 5 min and washed twice with PBS. The washed cells were resuspended in 1 mL PBS for imaging using a Nikon A1R confocal system (Thermo Fisher Scientific; Waltham, MA). The EGFP and mScarlet-I fluorescence was acquired using a 488 and 561 nm laser line and detected through a 530/30 and 585/42 nm bandpass emission filter, respectively. For analysis by flow cytometry, bacterial suspensions were diluted approximately 100-fold and analyzed using a CytoFLEX LX flow cytometer (Beckman Coulter; Brea, CA). For each sample, 50,000 events were collected for up to 10 min; doublets were excluded by using a forward scatter with 50 as the discriminator. Data was acquired using CytExpert 2.0 (Beckman Coulter; Brea, CA) and analyzed using FlowJo software [(v10.7), FlowJo LLC; Ashland, OR)]. All events were collected in R1 (Supplementary Fig6d_1_), and additional regions were defined for fluorescence analysis. Events in R2 and R3 (Supplementary Fig6d_2-_d_6_) were defined as bacteria expressing EGFP and mScarlet-I, respectively.

### Statistics

For sequencing data, the non-parametric Mann–Whitney U test was used to determine if differences were statistically significant in pairwise comparisons. Values are means ± SD. *p* < 0.05 was considered statistically significant. For metabolomics data, peak area values for metabolites were median scaled and log transformed, and any missing values were replaced with the minimal value detected in the data set. Log-transformed data were analyzed using a two-way analysis of variance (ANOVA) adjusted for multiple comparisons with the false discovery rate procedure with ArrayStudio/Jupyter Notebook and R package qvalue (version 2.20.0).^60^

### Reporting summary

Further information on research design is available in the Nature Research Reporting Summary linked to this article.

## Supplementary Material

Supplemental Material

## Data Availability

The data (raw reads) that support the findings of this study are openly available in NCBI BioProject at https://www.ncbi.nlm.nih.gov/bioproject, reference numbers PRJNA650546 (DNA) and PRJNA1086571 (RNA). The authors confirm that the metabolomics data supporting the findings of this study are available within the article and its supplementary materials.

## Code availability

Custom scripts for manipulating DNA sequencing outputs have been deposited on GitHub at https://github.com/publicationsubmission/naturecommunications2020.
