# Supplementary material for: Divergence of gut bacteria through the selection of genomic variants implicated in the metabolism of sugars, amino acids, and purines by small extracellular vesicles in milk
Source: Gut Microbes. 2025 Jan 6;17(1):2449704. doi: 10.1080/19490976.2025.2449704 (PMC12716043; doi:10.1080/19490976.2025.2449704)
Supplement: Supplemental Material [file KGMI_A_2449704_SM0357.zip › Supplementary file.docx]

**b**

**a**

**
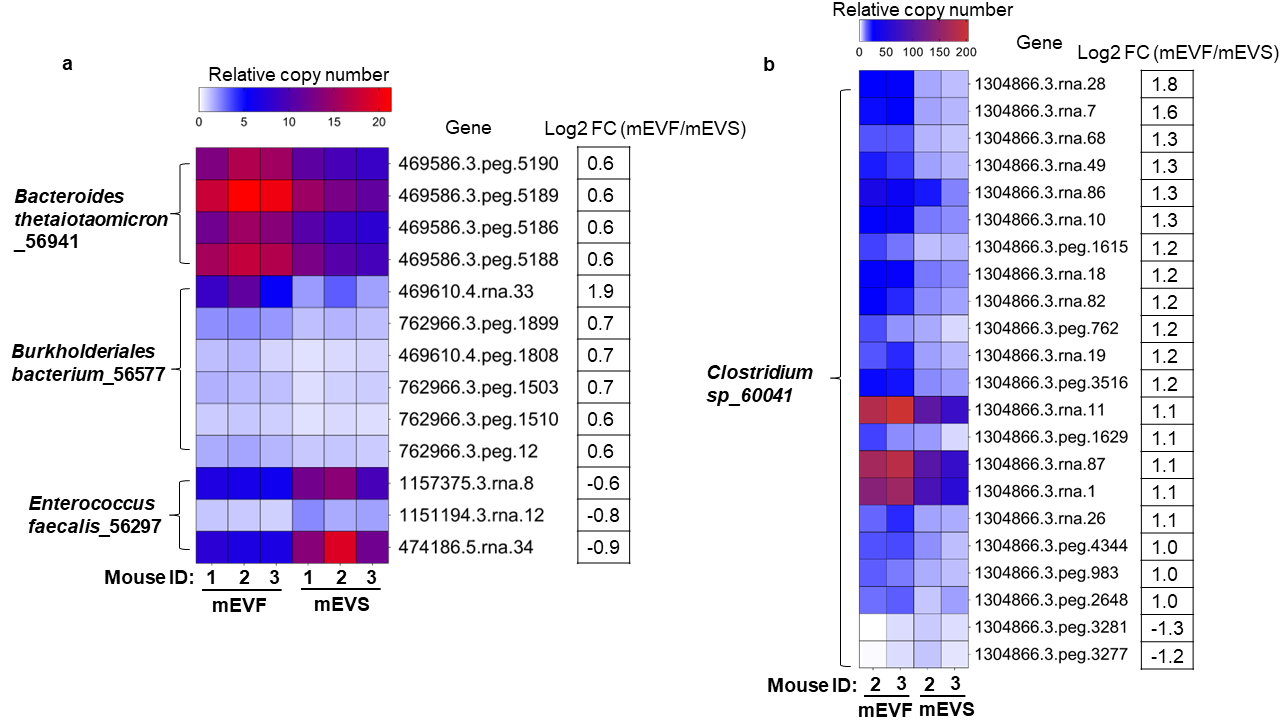
**

**Supplementary Figure 1. Relative copy number (RCN) of genes in bacteria cultured in media defined by their content of milk extracellular vesicles.**

Gut content was collected from the ceca of three C57BL/6J mice, aliquoted, and cultured in milk EVs (mEVS) or milk EV-free (mEVF) media under anerobic conditions for seven days. The relative copy number of genes were determined by using MIDAS. Columns represent mice and rows represent genes. **a** Relative copy numbers in genes detected in all biological replicates and in both culture conditions (*n* = 3, *p* < 0.05). **b** Relative copy number variations in *Clostridium sp._60041* detected in 2 out of 3 biological replicates. FC, fold change. Source data are provided as a Source Data file.

**
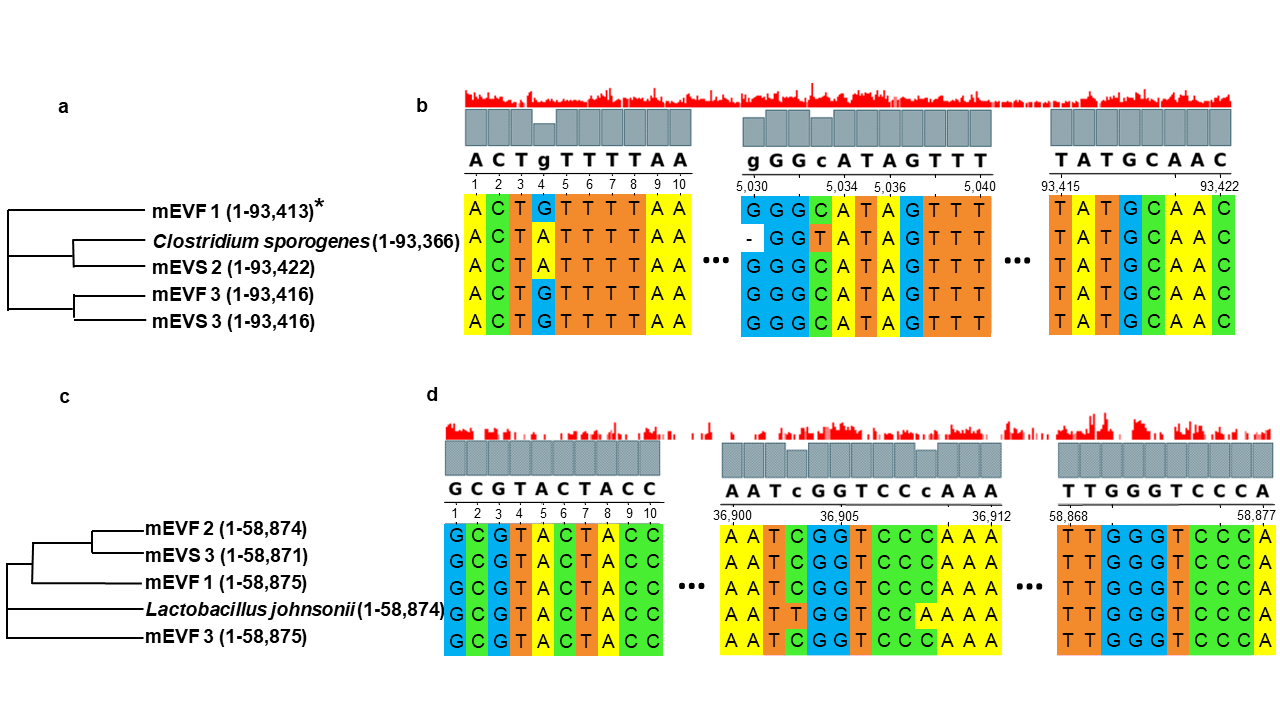
**

**c d**

**a b**

**Supplementary Figure 2. Genomic variations in *C. sporogenes* and *L. johnsonii* cultured in media defined by the content of milk extracellular vesicles.**

**a** Phylogenetic tree of *C. sporogenes* marker genes. **b** Multiple sequence alignment of concatenated *C. sporogenes* markers from mEVS and mEVF cultures compared to the *C. sporogenes* reference genome (RefSeq: GCF_000960175.1). **c** Phylogenetic tree of *L. johnsonii* marker genes. **d** Multiple sequence alignment of concatenated *L. johnsonii* markers from mEVS and mEVF cultures compared to the *L. johnsonii* reference genome (RefSeq: GCF_003316915.1). Gut content was collected from caeca of C57BL/6J mice and cultured in mEVS or mEVF media under anerobic conditions for seven days. The phylogenetic tree was built using the RAxML algorithm. *Numbers in parentheses identify the length of the concatenated markers. Sequencing reads of marker genes were aligned with the reference sequence in the MetaPhlAn2 database. Source data are provided as a Source Data file.


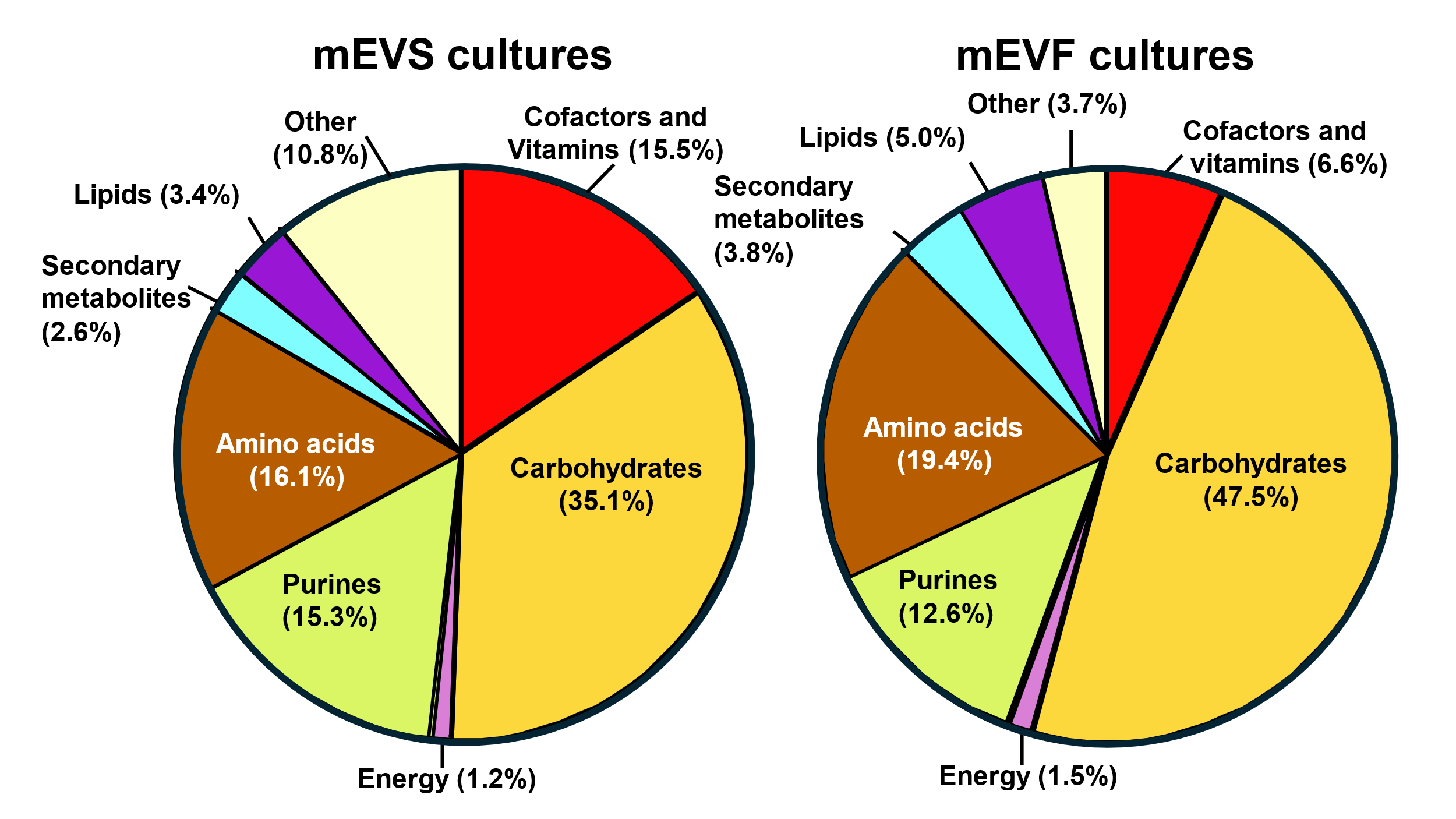


**Supplementary Figure 3. Metabolic pathways enriched for genomic variants in mEVS and mEVF cultures.** Variant transcripts were linked with metabolic pathways using the Bacterial and Viral Bioinformatics Resource Center database.

**Supplementary Figure 3.**

**Supplementary Figure 4.** **Schematic illustration of purine metabolism.** Xanthine is further hydroxylated to uric acid (the final product in primates) in cytosol. Uric acid is converted to allantoin in peroxisomes of other mammalian species.


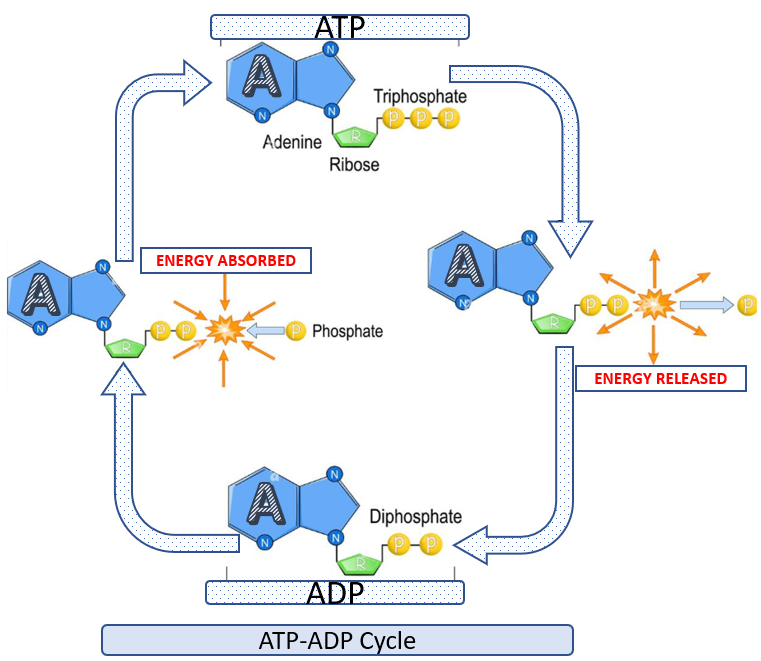

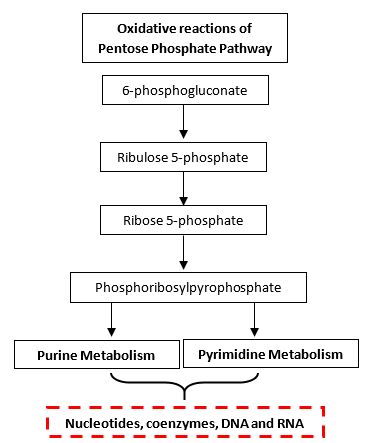

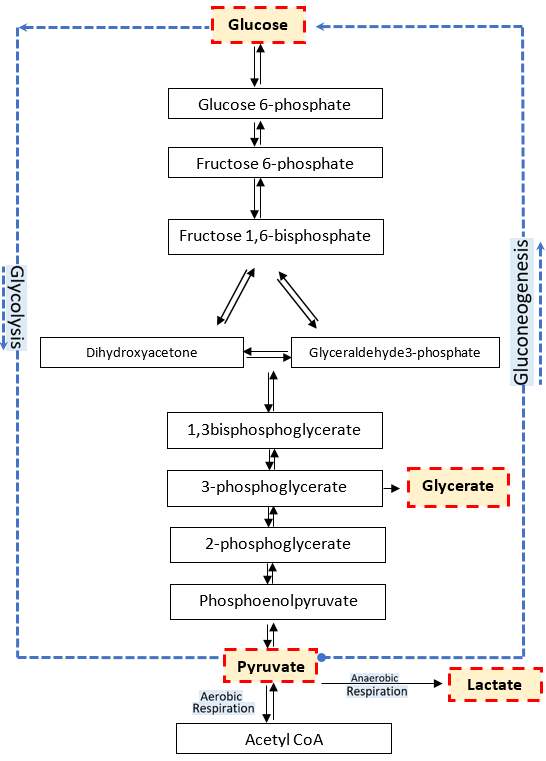

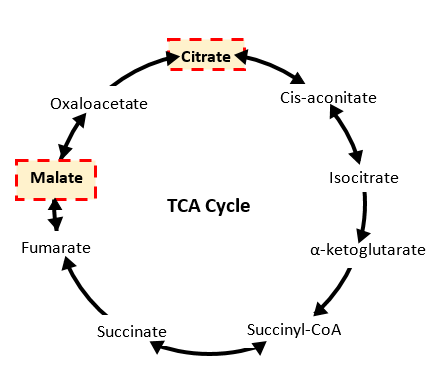


**High Energy electrons and H^+^**

**Electron transport chain**

**Supplementary Figure 5.** **Schematic representation of sugar and energy metabolism.**

This diagram illustrates the key pathways involved in the metabolism of sugars, highlighting glycolysis, the citric acid cycle (TCA cycle), and oxidative phosphorylation. The flow of energy is depicted, showing how glucose is converted into pyruvate and subsequently into ATP through various metabolic processes.

**Supplementary Figure 6.** **Internalization of milk extracellular vesicles by *Bacillus subtilis, Bifidobacterium infantis*, and *Escherichia coli*.**

**a** Time courses of HiLyte 750-labeled mEVs uptake by *B. subtilis* and *E. coli*. Data are expressed as mean ± SD (*n* = 3). **b** Bivariate plots of flow cytometric detection of delivery and expression of plasmid by mEVs to bacteria. (b_1_) Side scatter (SS) and forward scatter (FS) results of transformed bacteria in both strains. Cells within R1 were selected for fluorescence analysis in both strains. (b_2_) Control bacteria were negative for both green and red fluorescence. Bacteria in R2 and R3 were classified as positive for green fluorescence and red fluorescence, respectively (b_3-_ b_6_).


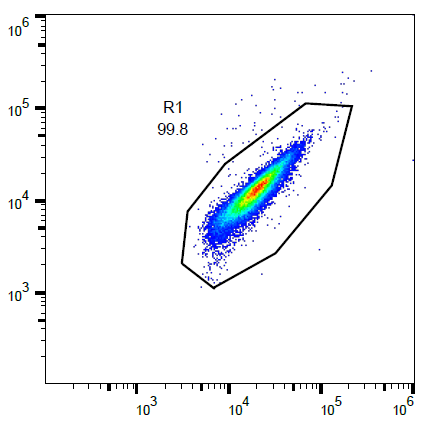


b_1_

SSC-A

FSC-A

All Events


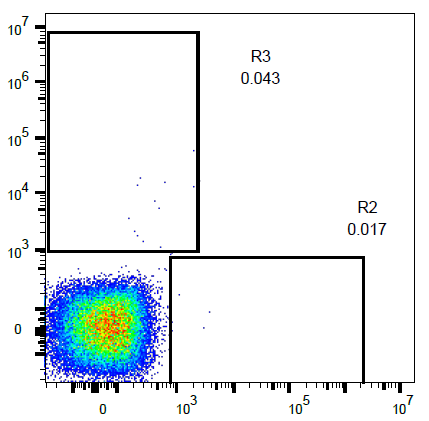


mScarlet-I YelFL1-A

Control

GFP BluFL1-A

b_2_

GFP BluFL1-A


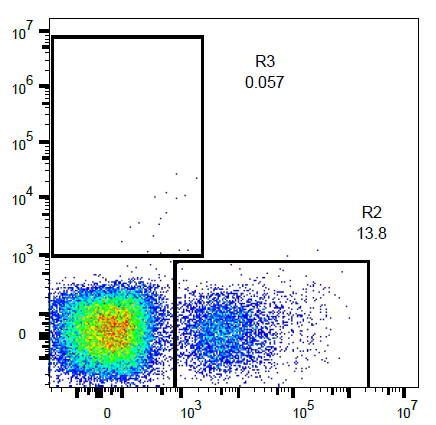


mScarlet-I YelFL1-A

*B. infantis_EGFP*

GFP BluFL1-A


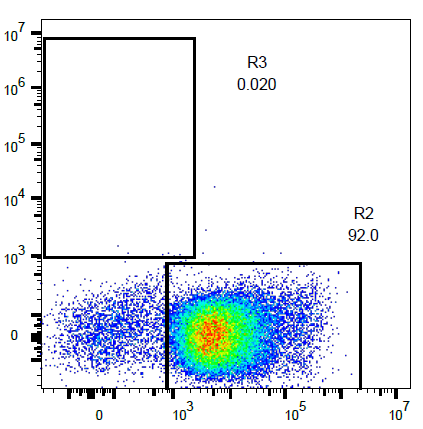


mScarlet-I YelFL1-A

*E. coli_EGFP*

b_3_

b_4_


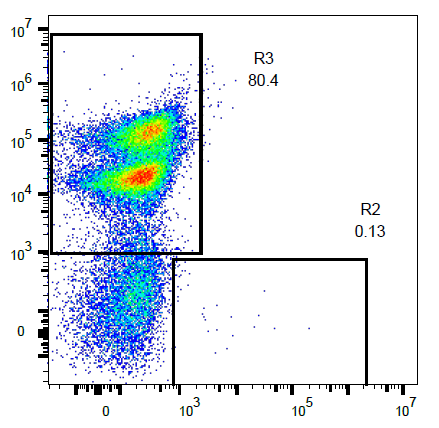


mScarlet-I YelFL1-A

*E. coli_mScarlet-I*

GFP BluFL1-A


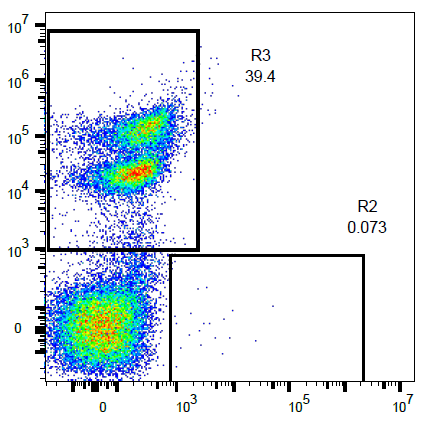


GFP BluFL1-A

mScarlet-I YelFL1-A

*B. infantis_mScarlet-I*

b_5_

b_6_


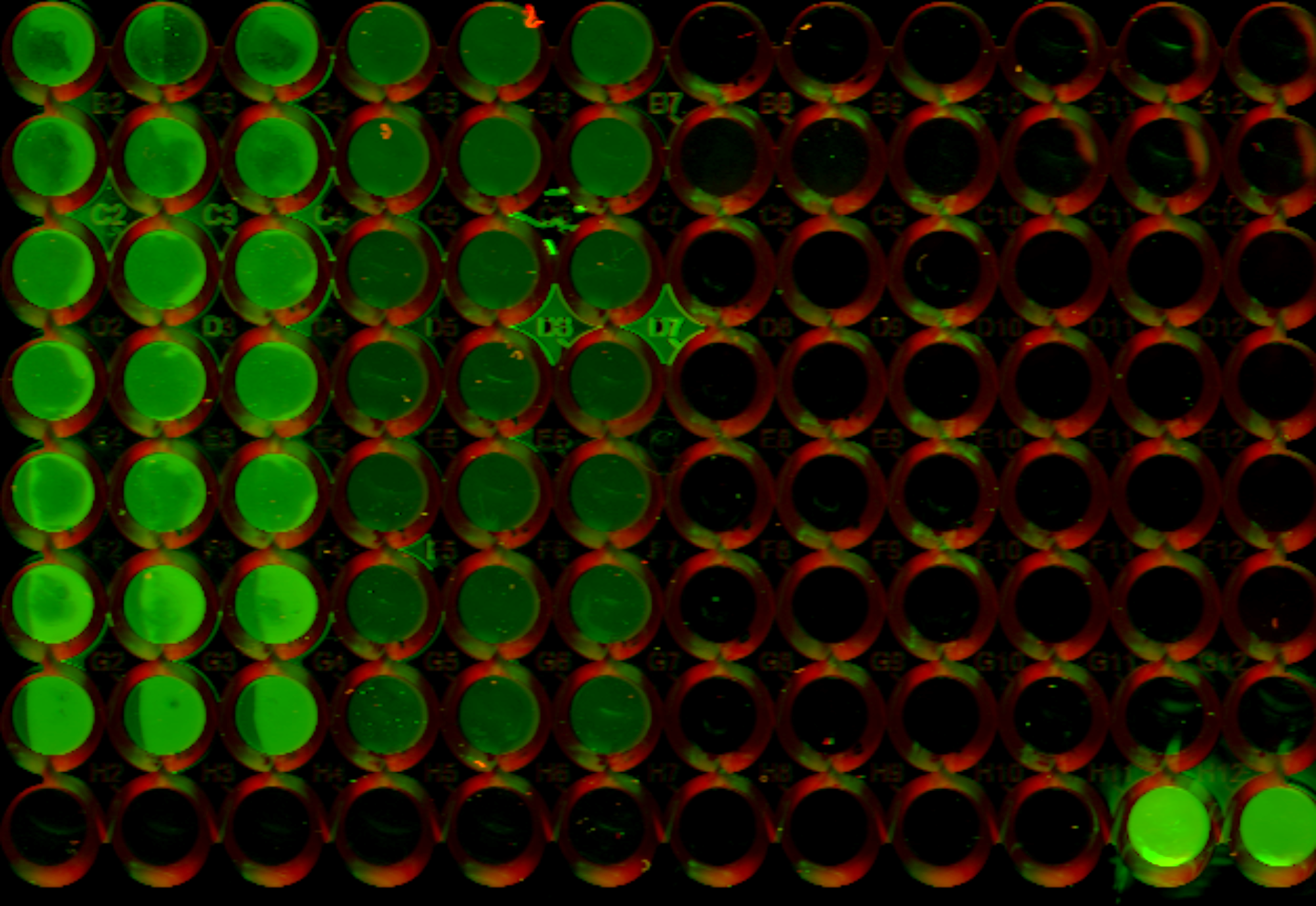

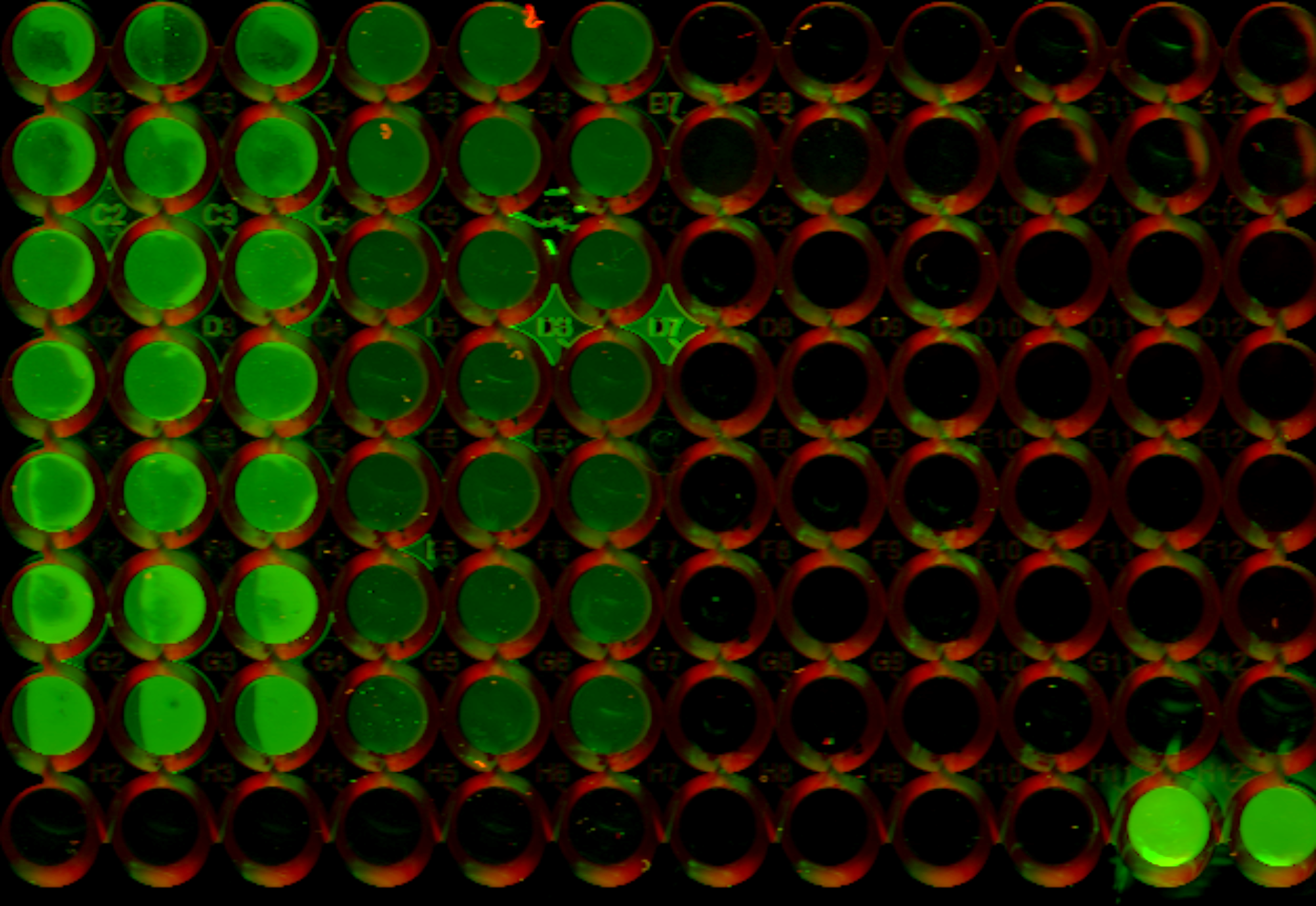


Pre

*B. subtilis*

Post


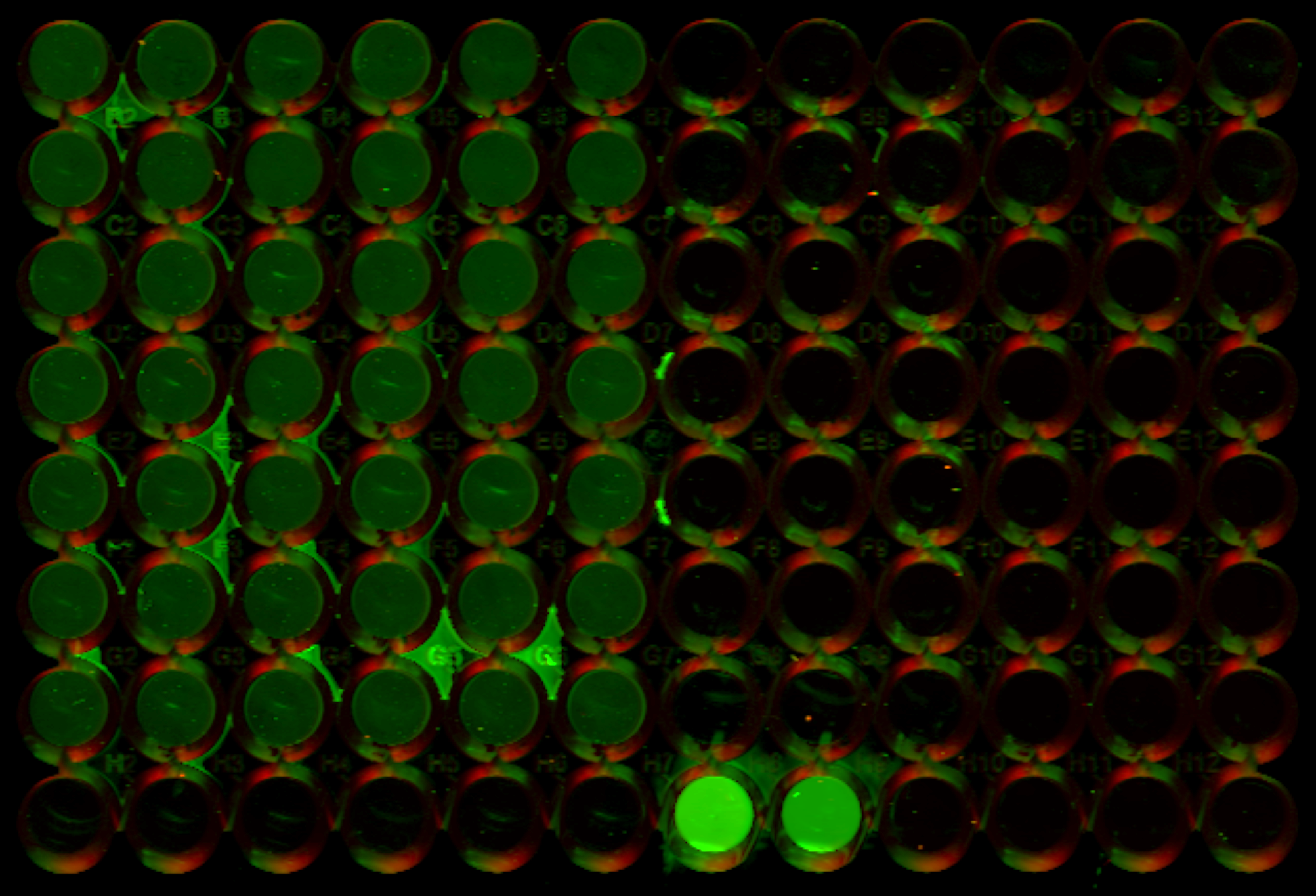

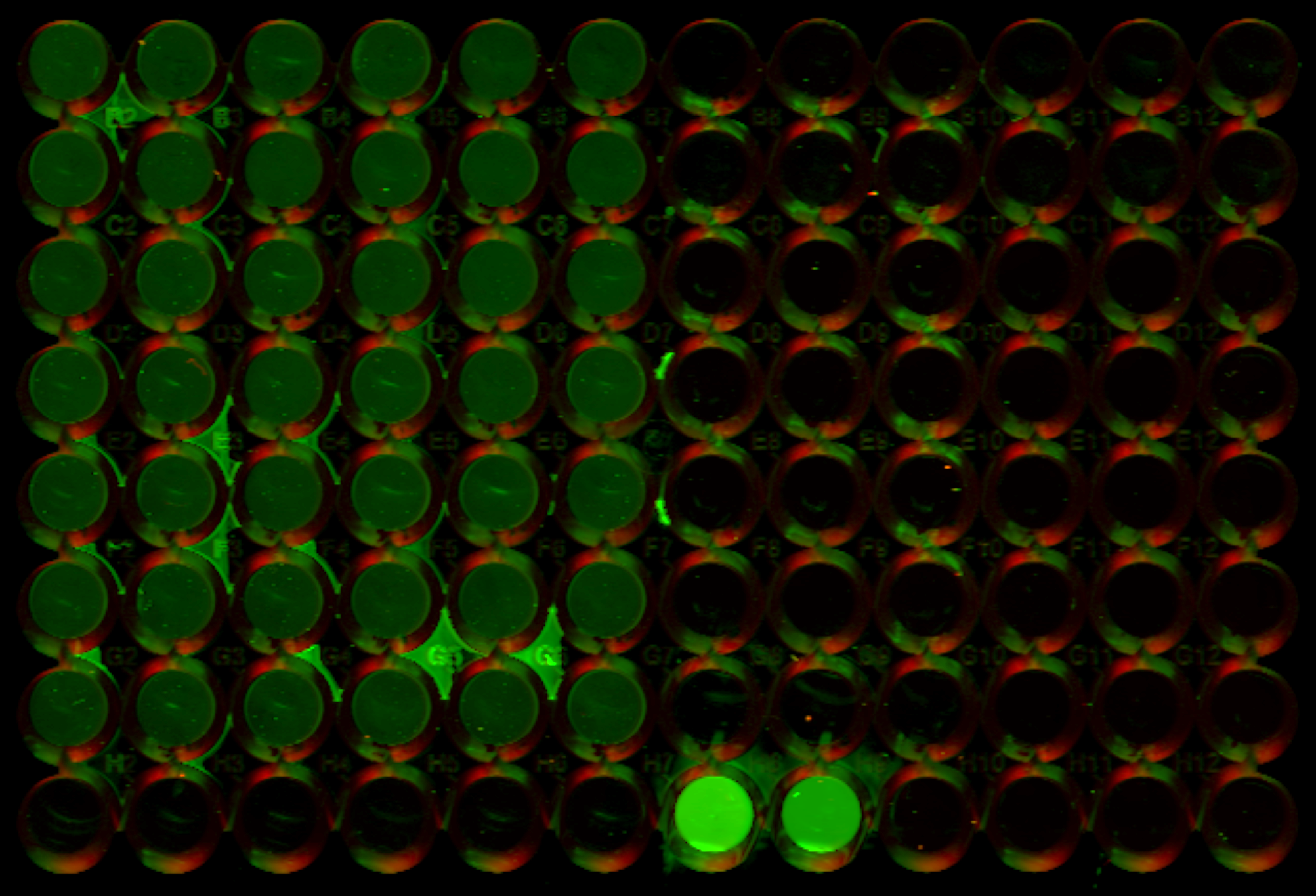


*E. coli*

Pre

Post

**a**

**b**

0

100

200

300

400

500

600

0

1000

2000

3000

*B. subtilis*

*E. coli*

Minutes

Fluorescence (arbitrary units)

**Supplementary Table 1. Summary of sequencing raw data.**

|  | **mEVS^a^** | **mEVF** |
| --- | --- | --- |
| Sequencing reads per sample^b^ | 127,935,309 ± 30,104,915 | 138,253,606 ± 25,740,862 |
| Total sequencing reads^c^ | 383,805,928 | 414,760,818 |
| Length of reads in bp  (minimum, maximum) | 35, 76 | 35, 76 |
| Length of reads (bp) | 73.0 ± 8.0 | 72.7 ± 8.4 |

^a^bp, base pairs; mEVF, milk extracellular vesicle-free media; mEVS, milk extracellular vesicle-supplemented media.

^b^Data are means ± SD (*n* =3).

^c^Sequencing reads from three biological repeats combined.

**Supplementary Table 2. Frequency of non-synonymous variants and afflicted metabolic pathways in bacteria, as identified by using MIDAS.**

| **Species** | **GenBank accession no.** | **Gene ID^a^** | **Biological repeat^b^** | | | **Metabolic pathway** |
| --- | --- | --- | --- | --- | --- | --- |
|  |  |  | **1** | **2** | **3** |  |
| **mEVF cultures^c^** | | | | | | |
| *E. faecalis_*56297 | AJAG01000020 | 1158976.3.peg.1478 | 27^d^ | 15 | 13 | ND |
| *E. faecalis_*56297 | AJAG01000037 | 1158976.3.peg.2969 | 186 | 238 | 105 | ND |
| *E. faecalis_*56297 | AJAG01000038 | 1158976.3.peg.3139 | 43 | 39 | 12 | ND |
| *E. faecalis_*56297 | AJAG01000019 | 1158976.3.peg.656 | 12 | 10 | 10 | ND |
| *C. celerecrescens_*61145 | JPME01000008 | 29354.3.peg.1203 | 26 | 18 | 35 | Glycolysis / Gluconeogenesis |
| *C. celerecrescens_*61145 | JPME01000008 | 29354.3.peg.1254 | 8 | 44 | 36 | ND |
| *C. celerecrescens_*61145 | JPME01000008 | 29354.3.peg.1259 | 6 | 16 | 27 | ND |
| *C. celerecrescens_*61145 | JPME01000010 | 29354.3.peg.1435 | 24 | 62 | 89 | Tryptophan metabolism |
| *C. celerecrescens_*61145 | JPME01000010 | 29354.3.peg.1482 | 12 | 23 | 41 | ND |
| *C. celerecrescens_*61145 | JPME01000010 | 29354.3.peg.1523 | 10 | 35 | 31 | ND |
| *C. celerecrescens_*61145 | JPME01000010 | 29354.3.peg.1620 | 11 | 24 | 32 | ND |
| *C. celerecrescens_*61145 | JPME01000010 | 29354.3.peg.1669 | 11 | 7 | 17 | ND |
| *C. celerecrescens_*61145 | JPME01000010 | 29354.3.peg.1689 | 15 | 56 | 87 | ND |
| *C. celerecrescens_*61145 | JPME01000011 | 29354.3.peg.1765 | 22 | 36 | 70 | Glycerophospholipid metabolism |
| *C. celerecrescens_*61145 | JPME01000011 | 29354.3.peg.1818 | 19 | 29 | 45 | ND |
| *C. celerecrescens_*61145 | JPME01000001 | 29354.3.peg.20 | 15 | 27 | 46 | Purine metabolism |
| *C. celerecrescens_*61145 | JPME01000013 | 29354.3.peg.2178 | 28 | 38 | 77 | ND |
| *C. celerecrescens_*61145 | JPME01000013 | 29354.3.peg.2245 | 15 | 38 | 68 | ND |
| *C. celerecrescens_*61145 | JPME01000014 | 29354.3.peg.2384 | 21 | 40 | 65 | ND |
| *C. celerecrescens_*61145 | JPME01000017 | 29354.3.peg.2899 | 14 | 35 | 61 | ND |
| *C. celerecrescens_*61145 | JPME01000018 | 29354.3.peg.3086 | 24 | 64 | 110 | ND |
| *C. celerecrescens_*61145 | JPME01000020 | 29354.3.peg.3308 | 15 | 42 | 47 | ND |
| *C. celerecrescens_*61145 | JPME01000022 | 29354.3.peg.3508 | 19 | 27 | 72 | ND |
| *C. celerecrescens_*61145 | JPME01000023 | 29354.3.peg.3568 | 17 | 43 | 68 | ND |
| **SUPPLEMENTARY TABLE 2, continued** | | | | | | |
|  |  |  |  |  |  |  |
| *C. celerecrescens_*61145 | JPME01000025 | 29354.3.peg.3723 | 22 | 48 | 64 | ND |
| *C. celerecrescens_*61145 | JPME01000002 | 29354.3.peg.38 | 21 | 48 | 70 | ND |
| *C. celerecrescens_*61145 | JPME01000026 | 29354.3.peg.3824 | 18 | 35 | 80 | ND |
| *C. celerecrescens_*61145 | JPME01000028 | 29354.3.peg.3921 | 17 | 29 | 49 | ND |
| *C. celerecrescens_*61145 | JPME01000028 | 29354.3.peg.3959 | 23 | 34 | 73 | ND |
| *C. celerecrescens_*61145 | JPME01000028 | 29354.3.peg.3965 | 9 | 30 | 70 | ND |
| *C. celerecrescens_*61145 | JPME01000003 | 29354.3.peg.420 | 20 | 20 | 105 | ND |
| *C. celerecrescens_*61145 | JPME01000035 | 29354.3.peg.4230 | 31 | 38 | 68 | ND |
| *C. celerecrescens_*61145 | JPME01000038 | 29354.3.peg.4342 | 10 | 16 | 20 | ND |
| *C. celerecrescens_*61145 | JPME01000046 | 29354.3.peg.4612 | 29 | 66 | 54 | ND |
| *C. celerecrescens_*61145 | JPME01000046 | 29354.3.peg.4615 | 22 | 52 | 73 | ND |
| *C. celerecrescens_*61145 | JPME01000003 | 29354.3.peg.484 | 19 | 34 | 73 | ND |
| *C. celerecrescens_*61145 | JPME01000003 | 29354.3.peg.586 | 24 | 38 | 60 | ND |
| *C. celerecrescens_*61145 | JPME01000002 | 29354.3.peg.59 | 19 | 46 | 64 | Arginine and proline metabolism |
| *C. celerecrescens_*61145 | JPME01000006 | 29354.3.peg.759 | 22 | 37 | 74 | ND |
| *C. celerecrescens_*61145 | JPME01000006 | 29354.3.peg.810 | 15 | 21 | 39 | ND |
| *C. celerecrescens_*61145 | JPME01000007 | 29354.3.peg.927 | 10 | 28 | 60 | ND |
| *C. celerecrescens_*61145 | JPME01000007 | 29354.3.peg.941 | 19 | 52 | 65 | ND |
|  |  |  |  |  |  |  |
| **mEVS cultures** | | | | | | |
|  |  |  |  |  |  |  |
| *C. celerecrescens_*61145 | JPME01000007 | 29354.3.peg.1023 | 55 | 62 | 113 | ND |
| *C. celerecrescens_*61145 | JPME01000008 | 29354.3.peg.1090 | 54 | 118 | 207 | ND |
| *C. celerecrescens_*61145 | JPME01000008 | 29354.3.peg.1125 | 49 | 39 | 94 | ND |
| *C. celerecrescens_*61145 | JPME01000008 | 29354.3.peg.1153 | 34 | 157 | 213 | ND |
| *C. celerecrescens_*61145 | JPME01000008 | 29354.3.peg.1282 | 47 | 144 | 269 | Starch and sucrose metabolism |
| *C. celerecrescens_*61145 | JPME01000009 | 29354.3.peg.1345 | 23 | 64 | 138 | ND |
| *C. celerecrescens_*61145 | JPME01000009 | 29354.3.peg.1346 | 28 | 85 | 218 | ND |
| *C. celerecrescens_*61145 | JPME01000010 | 29354.3.peg.1423 | 55 | 45 | 61 | ND |
| *C. celerecrescens_*61145 | JPME01000010 | 29354.3.peg.1434 | 42 | 98 | 182 | ND |
| **SUPPLEMENTARY TABLE 2, continued** | | | | | | |
|  | | | | | | |
| *C. celerecrescens_*61145 | JPME01000010 | 29354.3.peg.1486 | 62 | 131 | 54 | ND |
| *C. celerecrescens_*61145 | JPME01000010 | 29354.3.peg.1486 | 59 | 129 | 54 | ND |
| *C. celerecrescens_*61145 | JPME01000010 | 29354.3.peg.1505 | 19 | 76 | 21 | ND |
| *C. celerecrescens_*61145 | JPME01000010 | 29354.3.peg.1532 | 56 | 84 | 66 | ND |
| *C. celerecrescens_*61145 | JPME01000010 | 29354.3.peg.1597 | 54 | 195 | 260 | ND |
| *C. celerecrescens_*61145 | JPME01000010 | 29354.3.peg.1609 | 32 | 51 | 45 | ND |
| *C. celerecrescens_*61145 | JPME01000010 | 29354.3.peg.1643 | 51 | 39 | 203 | ND |
| *C. celerecrescens_*61145 | JPME01000010 | 29354.3.peg.1692 | 15 | 81 | 120 | ND |
| *C. celerecrescens_*61145 | JPME01000010 | 29354.3.peg.1707 | 27 | 15 | 10 | ND |
| *C. celerecrescens_*61145 | JPME01000010 | 29354.3.peg.1735 | 62 | 78 | 59 | ND |
| *C. celerecrescens_*61145 | JPME01000011 | 29354.3.peg.1809 | 42 | 96 | 266 | ND |
| *C. celerecrescens_*61145 | JPME01000011 | 29354.3.peg.1810 | 33 | 98 | 82 | ND |
| *C. celerecrescens*_61145 | JPME01000011 | 29354.3.peg.1815 | 46 | 115 | 242 | ND |
| *C. celerecrescens_*61145 | JPME01000011 | 29354.3.peg.1836 | 104 | 120 | 101 | ND |
| *C. celerecrescens_*61145 | JPME01000012 | 29354.3.peg.1907 | 30 | 87 | 163 | ND |
| *C. celerecrescens_*61145 | JPME01000012 | 29354.3.peg.1953 | 29 | 68 | 85 | ND |
| *C. celerecrescens_*61145 | JPME01000012 | 29354.3.peg.1953 | 32 | 108 | 151 | ND |
| *C. celerecrescens_*61145 | JPME01000012 | 29354.3.peg.1970 | 61 | 74 | 111 | Peptidoglycan biosynthesis |
| *C. celerecrescens_*61145 | JPME01000012 | 29354.3.peg.1976 | 36 | 70 | 68 | ND |
| *C. celerecrescens_*61145 | JPME01000012 | 29354.3.peg.2043 | 33 | 96 | 98 | Porphyrin and chlorophyll metabolism |
| *C. celerecrescens_*61145 | JPME01000012 | 29354.3.peg.2054 | 33 | 68 | 75 | ND |
| *C. celerecrescens_*61145 | JPME01000012 | 29354.3.peg.2075 | 58 | 73 | 206 | Porphyrin and chlorophyll metabolism |
| *C. celerecrescens_*61145 | JPME01000012 | 29354.3.peg.2091 | 19 | 61 | 132 | ND |
| *C. celerecrescens_*61145 | JPME01000012 | 29354.3.peg.2098 | 56 | 108 | 190 | ND |
| *C. celerecrescens_*61145 | JPME01000013 | 29354.3.peg.2179 | 58 | 136 | 197 | ND |
| *C. celerecrescens_*61145 | JPME01000013 | 29354.3.peg.2275 | 31 | 63 | 98 | ND |
| *C. celerecrescens_*61145 | JPME01000013 | 29354.3.peg.2276 | 45 | 43 | 62 | ND |
| *C. celerecrescens_*61145 | JPME01000013 | 29354.3.peg.2286 | 57 | 177 | 222 | ND |
| *C. celerecrescens_*61145 | JPME01000013 | 29354.3.peg.2359 | 73 | 105 | 118 | ND |
| *C. celerecrescens_*61145 | JPME01000013 | 29354.3.peg.2359 | 59 | 105 | 110 | ND |
| **SUPPLEMENTARY TABLE 2, continued** | | | | | | |
|  |  |  |  |  |  |  |
| *C. celerecrescens_*61145 | JPME01000013 | 29354.3.peg.2363 | 105 | 92 | 189 | ND |
| *C. celerecrescens_*61145 | JPME01000014 | 29354.3.peg.2509 | 37 | 110 | 170 | D-Glutamine and D-glutamate metabolism |
| *C. celerecrescens_*61145 | JPME01000015 | 29354.3.peg.2621 | 85 | 37 | 36 | Oxidative phosphorylation |
| *C. celerecrescens_*61145 | JPME01000015 | 29354.3.peg.2664 | 24 | 117 | 161 | ND |
| *C. celerecrescens*_61145 | JPME01000016 | 29354.3.peg.2858 | 49 | 132 | 164 | ND |
| *C. celerecrescens_*61145 | JPME01000002 | 29354.3.peg.289 | 30 | 162 | 83 | ND |
| *C. celerecrescens_*61145 | JPME01000018 | 29354.3.peg.2907 | 34 | 46 | 62 | ND |
| *C. celerecrescens_*61145 | JPME01000018 | 29354.3.peg.2907 | 27 | 96 | 111 | ND |
| *C. celerecrescens_*61145 | JPME01000018 | 29354.3.peg.2907 | 28 | 120 | 136 | ND |
| *C. celerecrescens_*61145 | JPME01000018 | 29354.3.peg.2907 | 42 | 159 | 222 | ND |
| *C. celerecrescens_*61145 | JPME01000018 | 29354.3.peg.2918 | 27 | 85 | 86 | ND |
| *C. celerecrescens*_61145 | JPME01000002 | 29354.3.peg.292 | 45 | 203 | 225 | Pantothenate and CoA biosynthesis |
| *C. celerecrescens*_61145 | JPME01000018 | 29354.3.peg.2999 | 53 | 51 | 227 | ND |
| *C. celerecrescens*_61145 | JPME01000018 | 29354.3.peg.3018 | 50 | 28 | 75 | Porphyrin and chlorophyll metabolism |
| *C. celerecrescens*_61145 | JPME01000018 | 29354.3.peg.3048 | 32 | 74 | 163 | ND |
| *C. celerecrescens_*61145 | JPME01000018 | 29354.3.peg.3060 | 54 | 79 | 112 | ND |
| *C. celerecrescens_*61145 | JPME01000020 | 29354.3.peg.3284 | 40 | 82 | 192 | ND |
| *C. celerecrescens_*61145 | JPME01000020 | 29354.3.peg.3308 | 55 | 102 | 199 | ND |
| *C. celerecrescens_*61145 | JPME01000020 | 29354.3.peg.3329 | 28 | 41 | 54 | ND |
| *C. celerecrescens_*61145 | JPME01000020 | 29354.3.peg.3368 | 60 | 156 | 87 | ND |
| *C. celerecrescens_*61145 | JPME01000020 | 29354.3.peg.3394 | 49 | 102 | 124 | ND |
| *C. celerecrescens_*61145 | JPME01000020 | 29354.3.peg.3404 | 48 | 123 | 121 | ND |
| *C. celerecrescens_*61145 | JPME01000024 | 29354.3.peg.3644 | 34 | 163 | 104 | ND |
| *C. celerecrescens_*61145 | JPME01000025 | 29354.3.peg.3662 | 39 | 82 | 203 | ND |
| *C. celerecrescens_*61145 | JPME01000025 | 29354.3.peg.3662 | 41 | 79 | 202 | ND |
| *C. celerecrescens_*61145 | JPME01000025 | 29354.3.peg.3702 | 29 | 163 | 249 | ND |
| *C. celerecrescens_*61145 | JPME01000025 | 29354.3.peg.3744 | 47 | 136 | 239 | ND |
| *C. celerecrescens_*61145 | JPME01000027 | 29354.3.peg.3874 | 79 | 88 | 226 | ND |
| *C. celerecrescens_*61145 | JPME01000028 | 29354.3.peg.3962 | 53 | 82 | 86 | ND |
| *C. celerecrescens_*61145 | JPME01000028 | 29354.3.peg.3962 | 53 | 86 | 74 | ND |
| **SUPPLEMENTARY TABLE 2, continued** | | | | | | |
|  |  |  |  |  |  |  |
| *C. celerecrescens_*61145 | JPME01000028 | 29354.3.peg.3972 | 81 | 208 | 221 | ND |
| *C. celerecrescens_*61145 | JPME01000031 | 29354.3.peg.4156 | 11 | 166 | 46 | ND |
| *C. celerecrescens_*61145 | JPME01000031 | 29354.3.peg.4166 | 13 | 7 | 48 | ND |
| *C. celerecrescens_*61145 | JPME01000003 | 29354.3.peg.419 | 55 | 77 | 222 | ND |
| *C. celerecrescens_*61145 | JPME01000002 | 29354.3.peg.42 | 80 | 127 | 165 | Amino sugar and nucleotide sugar metabolism |
| *C. celerecrescens_*61145 | JPME01000034 | 29354.3.peg.4223 | 66 | 130 | 151 | ND |
| *C. celerecrescens_*61145 | JPME01000035 | 29354.3.peg.4230 | 45 | 179 | 140 | ND |
| *C. celerecrescens_*61145 | JPME01000037 | 29354.3.peg.4304 | 77 | 58 | 39 | ND |
| *C. celerecrescens_*61145 | JPME01000038 | 29354.3.peg.4350 | 22 | 34 | 29 | Glycine, serine and threonine metabolism |
| *C. celerecrescens_*61145 | JPME01000039 | 29354.3.peg.4418 | 64 | 123 | 226 | ND |
| *C. celerecrescens_*61145 | JPME01000042 | 29354.3.peg.4499 | 40 | 157 | 140 | ND |
| *C. celerecrescens_*61145 | JPME01000044 | 29354.3.peg.4561 | 32 | 175 | 177 | ND |
| *C. celerecrescens_*61145 | JPME01000044 | 29354.3.peg.4569 | 25 | 82 | 100 | ND |
| *C. celerecrescens_*61145 | JPME01000047 | 29354.3.peg.4621 | 69 | 49 | 150 | ND |
| *C. celerecrescens_*61145 | JPME01000003 | 29354.3.peg.545 | 80 | 151 | 192 | ND |
| *C. celerecrescens_*61145 | JPME01000004 | 29354.3.peg.611 | 76 | 104 | 295 | ND |
| *C. celerecrescens_*61145 | JPME01000002 | 29354.3.peg.64 | 54 | 43 | 32 | Arginine and proline metabolism |
| *C. celerecrescens_*61145 | JPME01000004 | 29354.3.peg.643 | 71 | 100 | 211 | Amino sugar and nucleotide sugar metabolism |
| *C. celerecrescens_*61145 | JPME01000006 | 29354.3.peg.754 | 57 | 70 | 68 | ND |
| *C. celerecrescens_*61145 | JPME01000006 | 29354.3.peg.804 | 74 | 45 | 66 | ND |
| *C. celerecrescens_*61145 | JPME01000006 | 29354.3.peg.808 | 50 | 130 | 204 | ND |
| *C. celerecrescens_*61145 | JPME01000006 | 29354.3.peg.808 | 43 | 110 | 122 | ND |
| *C. celerecrescens_*61145 | JPME01000006 | 29354.3.peg.809 | 69 | 101 | 68 | ND |
| *C. celerecrescens_*61145 | JPME01000006 | 29354.3.peg.858 | 52 | 82 | 80 | Glycine, serine and threonine metabolism |
| *B. thetaiotaomicron_*56941 | NZ_GG695899 | 469586.3.peg.1027 | 72 | 64 | 11 | ND |
| *B. bacterium_*56577 | NZ_GL384006 | 469610.4.peg.2362 | 50 | 95 | 170 | ND |

^a^IDs as per the Pathosystems Resource Integration Center 3.6.8 (https://www.patricbrc.org/).

^b^Numerals denote the three mice used for collecting cecum content.

^c^mEVF, milk extracellular vesicle-free media; mEVS, milk extracellular vesicle-supplemented media.

^d^Numerals represent the number of genomic variations detected in each gene broken down by mouse and treatment. ND, not determined.

**Supplementary Table 3. Bacteria detected in both mEVS and mEVF cultures using StrainPhlAn.**

| **Species** |
| --- |
| *Aneurinibacillus aneurinilyticus* |
| *Anaerotruncus sp. G3* (2012) |
| *Bacillus cereus* |
| *Clostridium sporogenes*^a^ |
| *Desulfotomaculum ruminis* |
| *Enterococcus faecalis*^a^ |
| *Lactobacillus johnsonii*^a^ |
| *Oscillibacter sp.* 1-3 |

^a^Species with a minimal coverage of 50% of marker genes in StrainPhlAn analysis.

**Supplementary Table 4.** **Frequency of genomic variants in bacteria.^a^**

| **Species** | **Reference strain** | **Genbank accession** | **Entire genome** | **Protein coding loci** | |
| --- | --- | --- | --- | --- | --- |
|  |  |  |  | **Synonymous** | **Non-synonymous** |
| *C. sporogenes*^a^ | ASM96017v1_11579 | GCA_000960175.1 | 4554 | 655 | 3884 |
| *E. faecalis* | 4928STDY7071600 | GCA_902161805.1 | 971 | 552 | 419 |
| *L. johnsonii* | ASM331691v1_Byun-jo-01 | GCA_003316915.1 | 1190 | 305 | 879 |
| Total |  |  | 6715 | 1512 | 5182 |

^a^Combined total of genomic variations from all sequencing experiments in both mEVS and mEVF cultures. Genomic variations were analyzed using StrainPhlAn.

**Supplementary Table 5. Non-synonymous variants in protein-coding genes in *C. sporogenes*, *L. johnsonii* and *E. faecalis*.^a^**

| \| **NCBI Accession** \| **dbxrefs** \| \| --- \| --- \| | **Gene symbol** | **Gene product** | **Metabolic pathway** |
| --- | --- | --- | --- | --- | --- |
| ***C. sporogenes*** | | | |
| WP_040109320.1 | ND^b^ | Hypothetical protein 1 | ND |
| WP_003494793.1 | CLSPOx_RS17740 | S41 family peptidase | ND |
| WP_003361249.1 | NPD5_RS15965 | GntR family transcriptional regulator | ND |
| WP_052691626.1 | ND | Anti-sigma factor domain-containing protein | ND |
| WP_040108485.1 | ND | PLP-dependent aminotransferase family protein | ND |
| WP_046869736.1^c^ | ND | Murein biosynthesis integral membrane protein | ND |
| WP_003490194.1 | CLSPOx_RS05620 | Sodium : alanine symporter family protein | ND |
| WP_052691686.1 | ND | Hypothetical protein 2 | ND |
| WP_040109020.1 | ND | DUF5050 domain-containing protein | ND |
| WP_040108773.1 | ND | Sigma-70 family RNA polymerase sigma factor | ND |
| WP_080941269.1^c^ | ND | LamG domain-containing protein | ND |
| WP_003491564.1^d^ | CLSPOx_RS17375 | GIY-YIG nuclease family protein | ND |
| WP_046870096.1 | ND | MerR family transcriptional regulator | ND |
| WP_003491228.1^c^ | CLSPOx_RS08155 | YeeE/YedE family protein | ND |
| WP_033058086.1 | YyaC | Spore protease YyaC | ND |
| WP_003493610.1 | CLSPOx_RS09030 | S-adenosylmethionine decarboxylase proenzyme | ND |
| WP_003494562.1^d^ | CLSPOx_RS14525 | Hypothetical protein 3 | ND |
| WP_040109120.1 | ND | Nitrogenase iron-molybdenum cofactor biosynthesis protein | ND |
| WP_003491248.1^d^ | CLSPOx_RS18330 | NYN domain-containing protein | ND |
| WP_003494423.1^c^ | sigG | RNA polymerase sporulation sigma factor SigG | ND |
| WP_046870025.1^d^ | ND | Hypothetical protein 4 | ND |
| WP_040108450.1 | ND | HAD family hydrolase | ND |
| WP_003490244.1 | CLSPOx_RS05785 | Tetratricopeptide repeat protein | ND |
| WP_040108060.1^d^ | ND | ABC transporter ATP-binding protein | Two-component system |
| WP_040107966.1 | ND | TrkH family potassium uptake protein | ND |
| WP_003491083.1^c^ | uraA | Uracil permease | ND |
| WP_040108854.1 | ND | Isoleucine--tRNA ligase | ND |
| WP_003492030.1^c^ | CLSPOx_RS01255 | DegV family protein | ND |
| WP_040108502.1 | ND | HAMP domain-containing protein | Two-component system |
| WP_003496336.1^d^ | NPD5_RS00600 | Pilin | ND |
| WP_080315688.1 | CLSPOx_RS05965 | Amino acid ABC transporter substrate-binding protein | ND |
| **Supplementary Table 5, continued** | | | |
| WP_003496969.1 | CLSPOx_RS00225 | MFS transporter | Cationic antimicrobial peptide resistance |
| WP_003490913.1^d^ | CLSPOx_RS06980 | Lactate utilization protein | ND |
| WP_046869986.1^d^ | ND | Hypothetical protein 5 | ND |
| WP_033059526.1 | CLSPOx_RS09115 | Cation : proton antiporter | ND |
| WP_033059225.1 | CLSPOx_RS07960 | ABC transporter ATP-binding protein | ND |
| WP_003496578.1 | CLSPOx_RS11865 | CDP-alcohol phosphatidyltransferase family protein | ND |
| WP_003493082.1^d^ | CLSPOx_RS03190 | Hypothetical protein 6 | ND |
| WP_052690930.1 | ND | Hypothetical protein 7 | ND |
| WP_046870030.1^c^ | ND | Glycosyltransferase family 2 protein | ND |
| ***L. johnsonii*** | | | |
| WP_011161650.1 | LJ_RS02815 | AAA family ATPase | ND |
| WP_011161778.1^d^ | LJ_RS03610 | UDP-glucose-hexose-1-phosphate uridylyltransferase | Galactose metabolism |
| WP_011161962.1 | LJ_RS04920 | Hypothetical protein 8 | ND |
| WP_086874857.1^d^ | ND | Response regulator transcription factor | ND |
| WP_086874957.1 | ND | Choloylglycine hydrolase family protein | ND |
| WP_086875109.1^c^ | ND | Hypothetical protein 9 | ND |
| ***E. faecalis*** | | | |
| WP_002354838.1 | ND | WxL domain-containing protein | ND |
| WP_002356400.1 | SAW_RS13245 | Exonuclease | ND |
| WP_002358840.1 | ND | VOC family protein | ND |
| WP_002360041.1^c^ | SAW_RS09810 | PTS fructose transporter subunit IIA | ND |
| WP_002360048.1 | ND | Transcriptional regulator YeiL | ND |
| WP_002360056.1 | ND | LacI family DNA-binding transcriptional regulator | Pentose phosphate pathway |
| WP_002360121.1 | ND | DUF998 domain-containing protein | ND |
| WP_002361365.1 | ND | GrpB family protein | ND |
| WP_002383224.1 | SAW_RS15605 | MurR/RpiR family transcriptional regulator | ND |
| WP_002383772.1 | SAW_RS11105 | Helix-turn-helix domain-containing protein | ND |

^a^Genomic variations were analyzed by using StrainPhlAn.

^b^ND, not determined.

^c^Genomic variants detected only in mEVS cultures.

^d^Genomic variants detected only in mEVF cultures.

**Supplementary Table 6. KEGG pathways of genomic variants in *C. sporogenes*.^a^**

| **Media** | **Gene** | **Metabolic pathway^b^** |
| --- | --- | --- |
| mEVS | *add, ade, APRT, adk, apt, cyaB, purA, purB, purE* | Purine metabolism |
|  |  |  |
|  | *adk, btuR, coaD, cobU, metK, mog, nadA, nadD, purA, purB, thiF* | Biosynthesis of cofactors |
|  |  |  |
| mEVF | *trp52*, *trpS* | Aminoacyl-tRNA biosynthesis |

^a^Genetic variations were identified using StrainPhlAn.

**^b^***p* < 0.05 for all pathways shown.

**Supplementary Table 7. Transcription of genomic variants in bacteria.**

The table is provided as a separate attachment titled “Transcription of genomic variants in bacteria.xlsx” in Excel format. Source data are provided as a Source Data file.

**Supplementary Table 8. Summary metabolome.**

|  | **Metabolites^a^** | | **Metabolites increased in mEVS cultures** | |
| --- | --- | --- | --- | --- |
| **Super pathway** | Cells | Media | Cells | Media |
| Amino Acid | 39 | 63 | 12 | 23 |
| Carbohydrate | 4 | 8 | 1 | 4 |
| Cofactors and Vitamins | 6 | 6 | 3 | 2 |
| Energy | 1 | 3 | 0 | 1 |
| Lipid | 21 | 22 | 9 | 12 |
| Nucleotide | 2 | 5 | 2 | 3 |
| Peptide | 9 | 17 | 4 | 9 |
| Xenobiotics | 8 | 23 | 4 | 7 |
| Total | 90 | 147 | 35 | 61 |
| ^a^Significantly different in mEVS compared to mEVF cultures (*p* < 0.05; *n* = 3 independent cultures each). | | | | |

**Supplementary Table 9. Metabolites in bacterial pellets and culture media supernatants.** The table is provided as a separate attachment titled “Metabolites in bacterial pellets and culture media supernatants.xlsx” in Excel format.

**Supplementary Table 10. Composition of *Gut microbiota medium* (GMM).**

| **Component** | **Amount/L** | **Stock solution** |
| --- | --- | --- |
| Tryptone Peptone | 2 g |  |
| Yeast Extract | 1 g |  |
| Raffinose | 10 g |  |
| L-cysteine | 0.5 g |  |
| Meat Extract | 5 g |  |
| KH_2_PO_4_ | 100 mL | 1 M, pH 7.2 |
| MgSO_4_-7H_2_O | 0.002 g |  |
| NaHCO_3_ | 0.4 g |  |
| NaCl | 0.08 g |  |
| CaCl2 | 1 mL | 0.8 g/100 mL |
| Vitamin K (menadione) | 1 mL | 1 mg/mL |
| FeSO_4_ | 1 mL | 0.4 mg FeSO_4_/mL |
| Histidine Hematin Solution | 1 mL | 1.2 mg hematin/mL in 0.2 M histidine |
| Tween 80 | 2 mL | 25% |
| ATCC Vitamin Mix | 10 mL |  |
| ATCC Trace Mineral Mix | 10 mL |  |
| Resazurin | 4 mL | 0.25 mg/mL |
